# Special Issue: The Actin-Myosin Interaction in Muscle: Background and Overview

**DOI:** 10.3390/ijms20225715

**Published:** 2019-11-14

**Authors:** John Squire

**Affiliations:** 1 Muscle Contraction Group, School of Physiology, Pharmacology and Neuroscience, University of Bristol, Bristol BS8 1TD, UK; j.m.squire@bristol.ac.uk; Tel.: +44-7706-07-6383; 2 Department of Surgery and Cancer, Faculty of Medicine, Imperial College London, London SW7 2BZ, UK

**Keywords:** myosin filaments, actin filaments, the sarcomere, Z-band, M-band, myosin cross-bridge cycle, hypertrophic cardiomyopathy, dilated cardiomyopathy, rigor muscle, weak-binding state, strong-binding states, sarcomere compliance, myosin filament compliance, actin filament compliance, cross-bridge compliance, time-resolved X-ray diffraction, fluorescence methods, spin probe methods

## Abstract

Muscular contraction is a fundamental phenomenon in all animals; without it life as we know it would be impossible. The basic mechanism in muscle, including heart muscle, involves the interaction of the protein filaments myosin and actin. Motility in all cells is also partly based on similar interactions of actin filaments with non-muscle myosins. Early studies of muscle contraction have informed later studies of these cellular actin-myosin systems. In muscles, projections on the myosin filaments, the so-called myosin heads or cross-bridges, interact with the nearby actin filaments and, in a mechanism powered by ATP-hydrolysis, they move the actin filaments past them in a kind of cyclic rowing action to produce the macroscopic muscular movements of which we are all aware. In this special issue the papers and reviews address different aspects of the actin-myosin interaction in muscle as studied by a plethora of complementary techniques. The present overview provides a brief and elementary introduction to muscle structure and function and the techniques used to study it. It goes on to give more detailed descriptions of what is known about muscle components and the cross-bridge cycle using structural biology techniques, particularly protein crystallography, electron microscopy and X-ray diffraction. It then has a quick look at muscle mechanics and it summarises what can be learnt about how muscle works based on the other studies covered in the different papers in the special issue. A picture emerges of the main molecular steps involved in the force-producing process; steps that are also likely to be seen in non-muscle myosin interactions with cellular actin filaments. Finally, the remarkable advances made in studying the effects of mutations in the contractile assembly in causing specific muscle diseases, particularly those in heart muscle, are outlined and discussed.

## 1. Introduction—Nature’s Linear Motors

In human bodies and those of other animals there are beautifully designed molecular mechanisms which move our limbs, or pump our blood, or aid in peristalsis, and there are motile mechanisms in all cells that move cell organelles or other cargoes from one part of the cell to another. In all cases, molecules which are enzymes that can utilise the energy stored in adenosine triphosphate (ATP) move along molecular tracks. To produce muscular movement, the molecular tracks are actin filaments and the ATP-driven motors are myosin molecules [1]. In all non-muscle cells there are also actin filament tracks with myosin-like motors moving along them [2]. There are also other tracks, the microtubules, along which run ATP-driven motor proteins such as dynein and kinesin [2]. We will not discuss the microtubule-based system further here; our focus will be on the interaction of myosin molecules with actin filaments in muscle.

**Figure 1:**
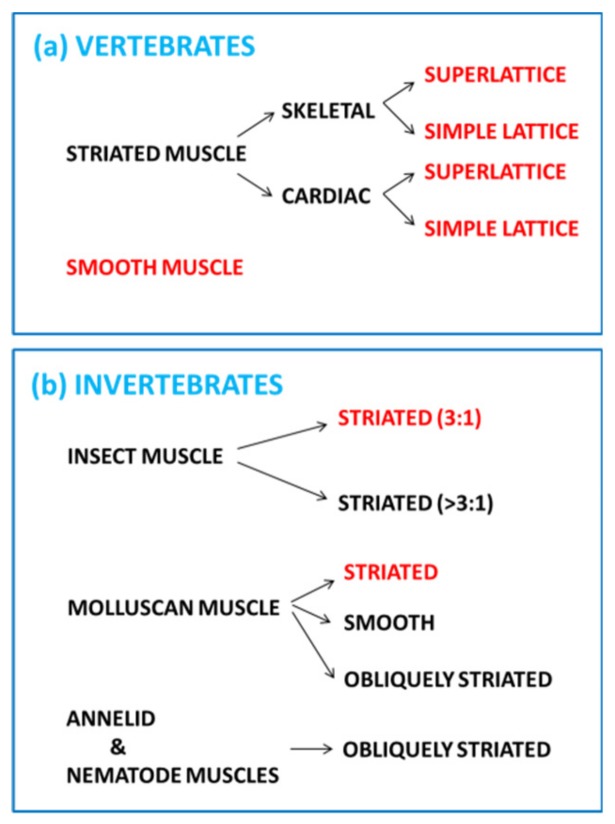
Summary of the main muscle types of (**a**) vertebrates and (**b**) invertebrates [3]. Muscle types which are commonly used in muscle research are highlighted in red. Vertebrate skeletal muscles occur in two distinct structural types, simple lattice and superlattice, but they are also distinguished physiologically in terms of different fibre types (e.g., fast, slow, intermediate). Insect muscles are classified in terms of the number of actin filaments that there are for each half myosin filament. Insect flight muscles often have 3 actins per half myosin filament, whereas leg muscles, for example, have more actins.

Animal bodies contain several distinctly different muscle types (Figure 1). Limbs and the skeleton are moved by striated muscles—they show transverse stripes with an axial separation between stripes of around 2 to 3 µm. Our skeletal muscles contain elongated multinucleate cells, called fibres, often about 10 to 100 µm in diameter and often many millimetres long. Muscle fibres contain cross-striated myofibrils, often about 1 to 10 µm in diameter and also very long. Our heart muscles are also cross-striated in a similar way to skeletal muscles, but the cells (cardiomyocytes), also containing myofibrils, are more like an irregular box and are generally much shorter than skeletal muscle fibres [3].

**Figure 2:**
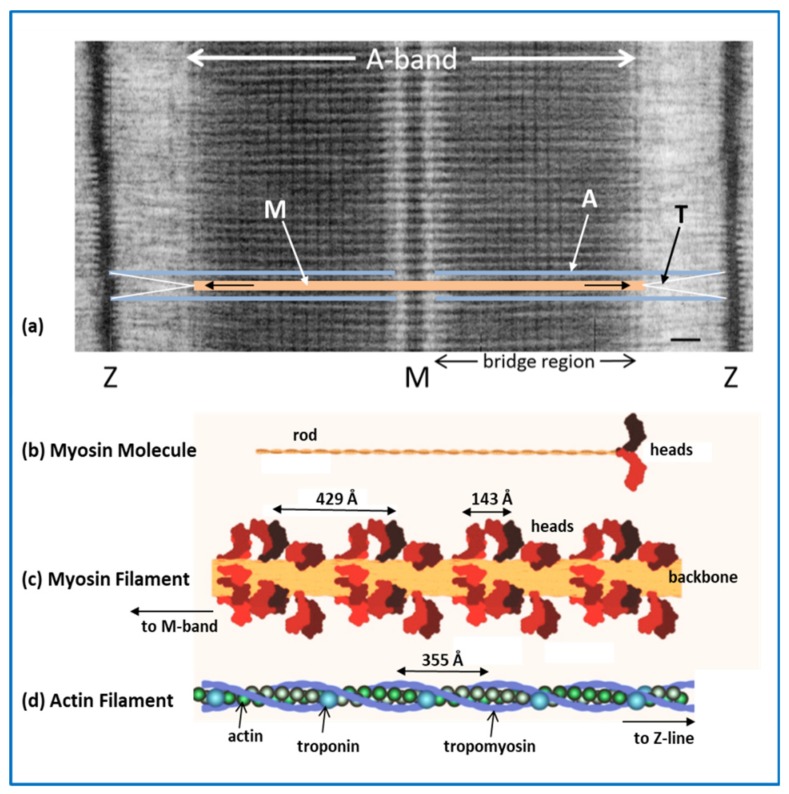
(**a**) Electron micrograph of a vertebrate striated muscle sarcomere (here about 2.3 µm long) which runs between two Z-lines (Z) to which actin filaments (A) are attached. The A-band contains myosin (thick) filaments (M), cross-linked at the M-band, and which the actin filaments partly overlap. (**b**) Simplified diagram of a myosin II molecule; a long rod on the end of which are two myosin heads which bind and hydrolyse ATP and bind actin. Myosin molecules (**b**) aggregate to form myosin filaments (**c**); the rods are in the backbone and the heads are almost helically arranged on the filament surface. The head ends of the rods, myosin S2 (subfragment-2) can lift from the filament surface to enable myosin head attachment to actin. Actin (thin-) filaments (**d**) comprise a twisted helical array of globular (G-)actin monomers along which run two strands of tropomyosin on which is also the troponin complex. For details see text. (Adapted from [7]).

As shown in the early 1950s in the classic work of Hugh Huxley and Hanson [4] and Andrew Huxley and Niedergerke [5], the striation pattern along a myofibril is due to overlapping sets of myosin filaments and actin filaments within each repeating unit (the sarcomere) as shown in Figure 2. When a muscle shortens these filament arrays slide past each other without the filaments themselves shortening very much (they do shorten very slightly as detailed later). This is the famous sliding filament model of muscle contraction. For a full description of these discoveries in the 1950s, their historical background, and brief biographies of the four authors see reference [6].

The other main muscle type in our bodies is smooth muscle. Vertebrate smooth muscles act on our intestines, our blood vessels and some other internal organs. Like other muscles, they contain actin and myosin filaments, but the myosin filaments are of a different type specialised to allow the muscles to shorten over a very large range of muscle lengths (see for example [8]). The muscles appear smooth because the myosin and actin filaments are not regularly organised into sarcomeres, although they do contain rudimentary contractile units with alternating myosin and actin filaments [8,9].

## 2. Striated Muscle Sarcomeres and the Contractile Mechanism

### 2.1. The Sarcomere

In Figure 2a, the sarcomere, from Z-line to Z-line, is part of a myofibril (horizontally-oriented in the Figure), which would be slightly wider than the vertical dimension at the scale in Figure 2a. As mentioned above, the sarcomere is the repeating unit or building block in striated muscles. To understand the molecular mechanisms involved in muscular contraction we need to know how the sarcomere works [3]. Since we know that the myosin and actin filaments slide past each other when the muscle shortens, we need to know what makes the filaments slide. We also need to know how muscles are turned on and off; skeletal muscles would not be much use if we couldn’t control them at will.

### 2.2. Myosin Filaments and the M-Band

**Figure 3:**
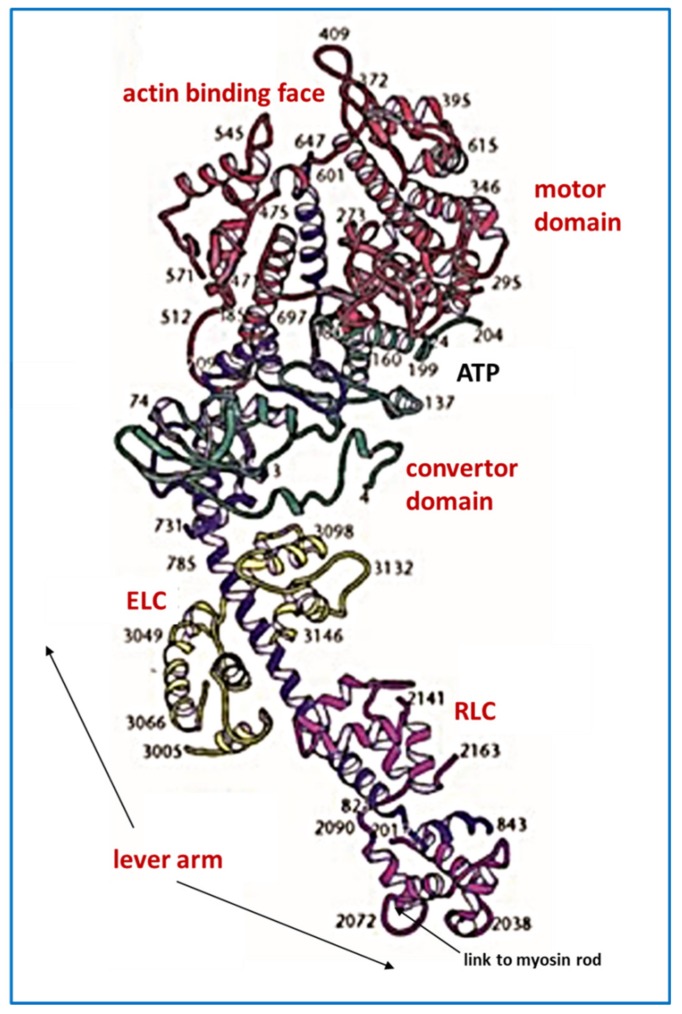
Structure of the myosin head from X-ray crystallography [12]. The top part is the motor domain, part of the myosin heavy chain, which can bind and hydrolyse ATP and can also bind to actin, which accelerates the ATPase. The lower part is the lever arm. It contains a central α–helix, also, part of the heavy chain, around which are wrapped two light chains, the essential light chain (ELC, yellow) and the regulatory light chain (RLC, maroon). Between the motor and the lever arm is the converter domain, which can be thought of as a kind of gear box. In the muscle contractile cycle the motor domain binds to actin, products of hydrolysis (ADP and Pi) are released and the lever arm tends to rotate around the converter domain; it will swing to a new position if it is free to do so, or provide a constant force if it is restrained and the lever arm cannot move. Adapted from [7].

Figure 2b shows the structure of a myosin II molecule as found in muscle. There is a whole family of myosins, most of which are involved in cell motility (see [10]). Muscle myosin is myosin II (Roman numeral for 2). It consists of a long 2-chain coiled-coil α–helical rod, about 1500 Å long and 20 Å in diameter (see [3,11]), on the end of which are two globular domains, the myosin heads. The chain in each coiled-coil in the rod continues into the myosin head, which also has two so-called light chains associated with it, the essential light chain (ELC) and the regulatory light chain (RLC) [11]. After the rod (Figure 3), comes the lever arm where the main chain forms a long α–helix on which are wrapped the two light chains. The main chain then continues through the converter domain into the motor domain. This has the ATP binding site and the actin binding face, and is the enzymatic part of the myosin molecule. It hydrolyses ATP to Adenosine diphosphate (ADP) and inorganic phosphate (Pi). The release of these products is activated by actin binding (see below).

**Figure 4:**
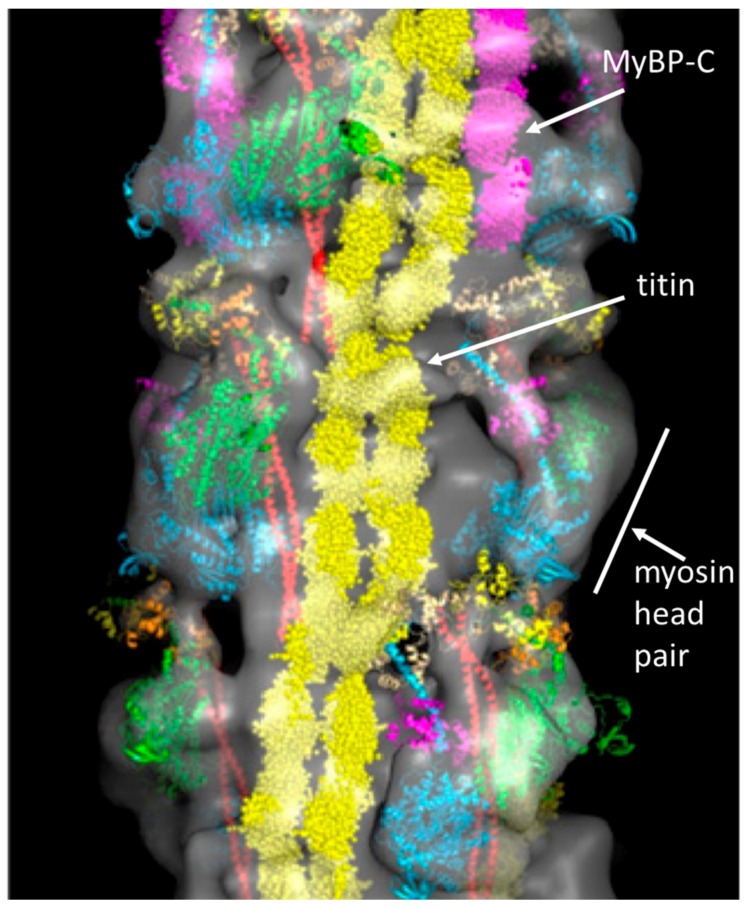
3D reconstruction of part of the bridge region of myosin filaments from human cardiac muscle [20]. The image shows a length of about 450 Å, containing three crowns of head pairs. The possible location of strands of titin and the accessory protein C-protein (MyBP-C) are shown in yellow and mauve respectively. The myosin head densities have been fitted with interacting head motif structures ([21]; discussed later). This conformation is supposed to be the position of the heads in what has been called the super-relaxed state when the ATP turnover rate is very low (see Section 2.4). Adapted from [20].

Myosin filaments are formed by the aggregation of myosin molecules. The myosin rods pack together with a regular axial stagger in the backbone of the myosin filament, and the myosin heads are located on the filament surface. The way the rods pack together was proposed in 1963 [13], but not confirmed for insect flight muscle myosin filaments until 2016 [14]. Vertebrate striated muscle myosin filaments are bipolar filaments; the rods pack together in an anti-parallel way in the middle of the filament (near the M-band) and in a parallel fashion on both sides of this, through the rest of the A-band. So there is a region of filament backbone in the middle (the bare zone) which is free from projecting myosin heads. Elsewhere, in the bridge regions, the heads project out from the filament surface in a quasi-helical array (Figure 2c and Figure 4), giving a structure which repeats almost exactly after 429 Å. Vertebrate striated muscle myosin filaments have 3-fold rotational symmetry [15,16,17], with the heads of three myosin molecules projecting out at the same axial position along the filament, but rotated around the backbone by multiples of 120°. One such set of three 120°-spaced head pairs is called a ‘crown of heads’. There are three such crowns within the 429 Å repeat, but successive crowns are rotated around the filament axis by roughly 40°. The axial separation of crowns is approximately 429/3 = 143 Å, but there is a systematic variation (or perturbation) in this [18,19].

In the A-band the myosin filaments form a hexagonal lattice when the sarcomere is viewed in cross-section (see Figure 2b of ref [22]). The distance between myosin filaments is around 400 to 450 Å, depending on the sarcomere length (i.e., the amount of filament overlap) The filaments are crosslinked at the M-band principally by the M-band proteins myomesin, creatine kinase and M-protein [23,24,25]. 

These M-band interactions appear to be sufficient to define whether the muscle has a superlattice or simple lattice structure (Figure 1 [26,27]). In simple lattice A-bands the myosin filaments all have the same rotation around their long axes, whereas in superlattice muscles the myosin filaments can have one of two rotations 60° apart, but the distribution of these rotations is not completely regular. Interestingly, it is the muscles of higher vertebrates (e.g., rabbit, chicken, frogs, humans) that have the relatively irregular superlattice structure, whereas the regular simple lattice is found mainly in the muscles of bony fish [28]. Superimposed on these two types of A-band structure, there are subtle variations in contractile protein isoforms, M-band structure, Z-band structure and energy supply (amongst other things) which give vertebrate striated muscle fibres different physiological properties; there are fast, slow and intermediate fibre types [29].

There is a direct link between the ends of the myosin filaments and the Z-line through the giant elastic protein titin (Figure 2a [28,30,31,32,33]). Apart from anything else, the titin molecules provide a restoring force when the sarcomere is stretched, so that the A-band tends to stay in the middle of the sarcomere. Titin molecules run along the whole of each half myosin filament from the M-band along the bridge region, to form so-called end-filaments at the myosin filament tips [32], and then through the I-band to the Z-line [33]. Different fibre types may have different titin isoforms, which give them different elastic properties [31]. Myosin filaments are also labelled in the central third of each bridge region by C-protein (Myosin Binding Protein-C; MyBP-C; Figure 4) which can have structural and regulatory roles [34,35,36].

### 2.3. Actin Filaments and the Z-Line 

**Figure 5:**
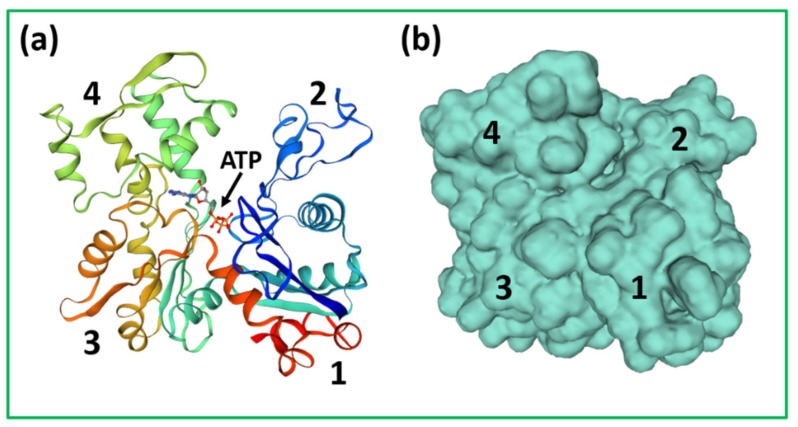
Structure of the G-actin monomer [37], but here from human cardiac muscle (3DAW). (**a**) Ribbon diagram and (**b**) surface representation of G-actin showing the four sub-domains and the central ATP-binding pocket. In actin filaments (e.g., Figure 2d or Figure 9a) sub-domains 3 and 4 lie on the inside where they interact with sub-domains 3 and 4 of neighbouring actin monomers, and sub-domains 1 and 2 lie on the outside of the filament. Myosin heads bind preferentially to sub-domain 1. Generated using the Swiss-Prot database [48].

Actin (thin) filaments are composed mainly of G-actin monomers ([37]: Figure 5) that have aggregated to form long helical assemblies (filamentous actin; F-actin [38]) along which run strands of the regulatory proteins tropomyosin and troponin (Figure 2d). Tropomyosin molecules are rather like part of the myosin rod in that they are parallel 2-chain α–helical molecules, but they are only about 400 Å long [3,39,40,41]. Tropomyosin molecules link end-to-end to form long strands along the actin filaments; they follow the long-pitched helical symmetry of the actin filaments. In relaxed muscles (ATP present, but very low calcium concentrations) the tropomyosin strands interact with the outer sub-domains 1 and 2 of actin. Each tropomyosin molecule interacts with seven actin monomers, and also binds one troponin complex [39]. The troponin complex has three components, troponin-C (Tn-C), which reversibly binds calcium ions in the physiological range, troponin-I (Tn-I), which is inhibitory, and troponin T, which interacts with tropomyosin [3,39,42,43]. For more details of thin filaments see reference [39].

In the vertebrate striated muscle sarcomere the actin filaments, which are about 1 µm long, are linked through the roughly square Z-line to actin filaments in the next sarcomere. The actin filament arrays on each side of the Z-line therefore point in opposite directions, just as the myosin filament arrays in the bridge regions point in opposite directions (or have opposite polarity) on each side of the M-band [3]. The cross-linking structure in the Z-band is primarily the protein α–actinin [44,45], although the Z-line part of titin and other proteins are also involved [33,46,47]. 

### 2.4. The Muscle Resting State

**Figure 6:**
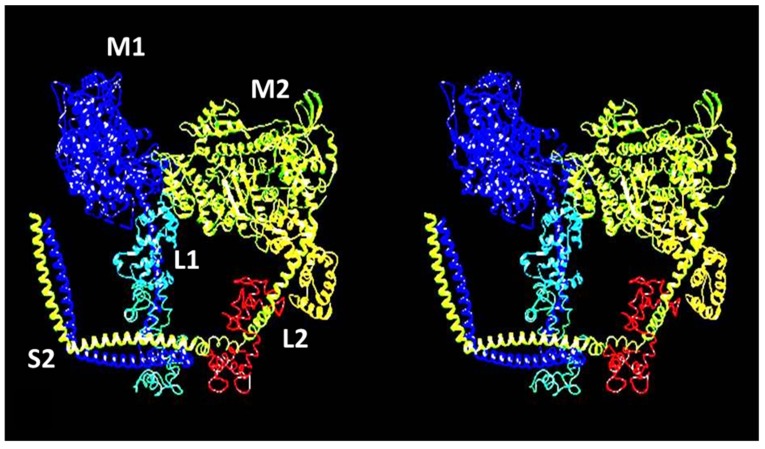
Wall-eyed stereo image of the interacting head motif structure seen on most relaxed myosin filaments (see Figure 4). Here M represents the motor domains and L represents the lever arms. The two lever arms come together at the first part of the coiled-coil myosin rod, known as subfragment-2 or S2. The motor domain (M2) of head 2 interacts with the M1 motor domain through its actin-binding site. Head M2 is therefore referred to as the docked head. The other head (M1), where the actin binding site is exposed, is termed the free head.

The image of the human cardiac muscle myosin filament structure in Figure 4 has the myosin head pairs in what is known as the interacting heads motif (IHM) structure [21]. This is illustrated in Figure 6 and is thought to be a resting state of the heads in which the usage of ATP is minimised. Myosin filaments with this structure are said to be in the super-relaxed state [49,50]. This kind of head interaction was first seen in 2D crystals of vertebrate smooth muscle myosin [21], but was then found on all types of relaxed myosin filament [20,51,52,53,54] when studied at high enough resolution by electron microscopy and single particle analysis (see Figure 4). 

### 2.5. The Contractile Cycle

**Figure 7:**
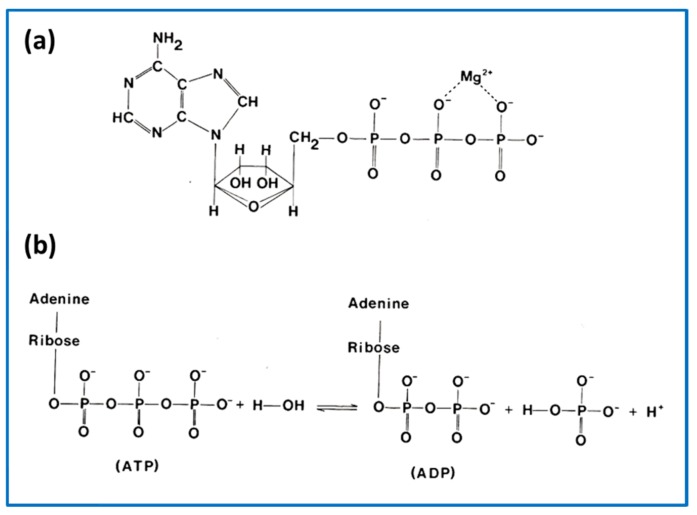
(**a**) Structure of the ATP molecule showing the adenine group, the ribose ring, and the three terminal phosphate groups. Also indicated is the position of the chelated magnesium ion on the last two phosphates. This is the normal Mg-ATP form of ATP found in muscle. (**b**) Structural representation of the ATP hydrolysis reaction in which ADP and inorganic phosphate (Pi) are the products. Reproduced from [3].

Now that we have all the main protagonists in play we can think more about the contractile cycle itself. First of all, as mentioned above, the contractile cycle is powered by the hydrolysis of ATP in the reaction ATP + H_2_O → ADP + Pi. The structure of ATP and details of the hydrolysis reaction are shown in Figure 7. 

**Figure 8:**
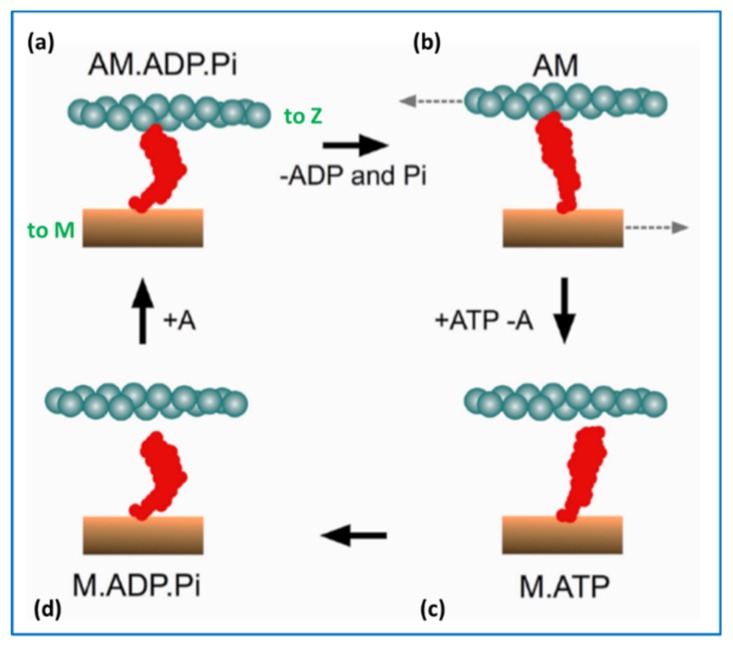
A simplified ATP-driven contractile cycle according to the scheme of Lymn and Taylor [55]. For details see text. Adapted from [3]. Red, myosin head; brown, myosin filament backbone, blue/green actin filament. (**a**) initial attached state, (**b**) end state (rigor-like) after product (ADP and Pi) release, (**c**) detached state induced by ATP binding, (**d**) myosin head after ATP hydrolysis with products ADP and Pi bound.

In the muscle contractile cycle (Figure 8), ATP bound to the myosin head (M.ATP;(c)) is hydrolysed to M.ADP.Pi (d) which can then bind to actin (a) to give AM.ADP.Pi. Actin attachment accelerates the release of products Pi and then ADP to give AM (b). AM is the rigor state, which occurs transiently in the contractile cycle, but relatively permanently in rigor mortis when, after death, ATP production in the body has stopped. This explains why in rigor mortis the cross-linked muscles are stiff. In the contractile cycle, heads in the AM state bind ATP, which accelerates detachment of heads from actin to give M.ATP and the hydrolysis cycle can start again. These various steps are associated with different myosin head shapes. 

The product release step (a) to (b) is thought to produce a rotation of the lever arm and hence relative axial movement of the actin and myosin filaments if they are free to move. The transition from (c) to (d) is a recovery step when the motor domain reverts to its original angle on the lever arm.

An interesting, and fundamentally important, observation by Bernhard Brenner and his colleagues [56] was that, even in relaxed vertebrate striated muscles, if the ionic strength of the bathing medium in the muscle is lowered, then the myosin heads can still bind transiently to actin in what is called the weak-binding state. Normal physiological ionic strength in muscles is around 170 mM, and Brenner et al. studied rabbit muscle fibres with ionic strengths reduced to as low as 20 mM. The weak-binding state is a very rapid equilibrium between the M.ADP.Pi state and what we will call the (A)M.ADP.Pi state; the brackets signify weak-binding to actin. Any sort of myosin head attachment to actin will increase the stiffness of the sarcomere, as we discuss below. An interesting property of the weak binding heads is that the apparent stiffness of the sarcomere depends on how fast the sarcomere is stretched. The faster the stretch, the more heads are trapped in transient attachments and the higher is the muscle stiffness. 

The weak binding state was later found to be present not only in low-ionic strength bathing solutions, but also as part of the normal contractile cycle [57,58,59]. So the step (d) to (a) in the cycle in Figure 8 is actually at least two steps: M.ADP.Pi to (A)M.ADP.Pi to AM.ADP.Pi, where the first step is the weak-binding/ rapid equilibrium step and AM.ADP.Pi is the first strongly-attached state. States AM.ADP and AM are also strong states. 

### 2.6. Muscle Regulation

Turning now to how we control our muscles, an early finding was that muscles are activated by the release of calcium ions (Ca^2+^) from calcium stores in the membranous sarcoplasmic reticulum surrounding muscle myofibrils [3,60,61]. This release is triggered by nerve signals from the brain travelling through our motor nerves to the fibre membrane, the sarcolemma, which becomes depolarised [3]. From there the signals are propagated towards the interior of the muscle fibres along T-tubules, which course their way through to all the myofibrils. Simultaneous depolarisation throughout the fibre activates the whole fibre as one unit. 

**Figure 9:**
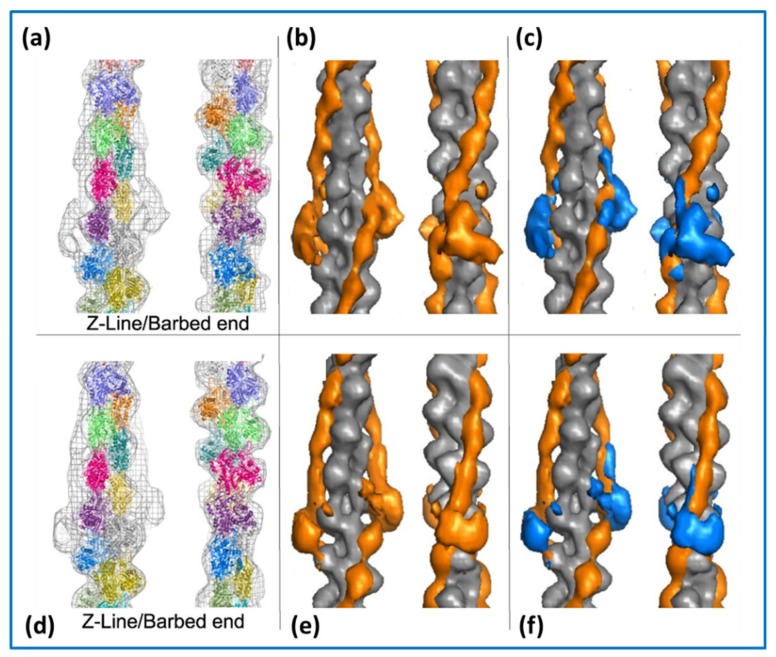
3D reconstructions of thin filaments with and without Ca^2+^ bound [39]. (**a**–**c**) Data from Ca^2+^-treated filaments, (**d**–**f**) data from Ca^2+^-free filaments. (**a**,**d**) Wire mesh representation of the single particle based reconstructions of the thin filament in the two states. An atomic F-actin model [38] is docked into the reconstruction and each subunit is colour coded. The barbed end (Z-line end) of the actin filament is at the bottom of the figure. (**b**,**e**) Difference density maps calculated by subtracting the docked F-actin model (grey) from the single particle reconstructions. This leaves the density attributable to the regulatory proteins troponin and tropomyosin (both orange). (**c**,**f**) Difference density maps calculated by subtracting docked F-actin (grey) and tropomyosin (orange) models from the single particle reconstructions leaving density attributable only to troponin (blue). Reproduced from [39] with permission.

Once Ca^2+^ has been released into the myofibrils it binds to troponin-C on the actin filaments, which alters the structure of troponin thereby causing the tropomyosin strands to which it is attached to move across the face of the actin filament. This can be seen in Figure 9 where the top images are of the actin filament with Ca^2+^ bound and the lower images are of calcium-free actin filaments. The shift of tropomyosin when calcium binds uncovers or modifies the site on sub-domain 1 of actin to which the myosin heads need to bind. The heads then bind to actin and the contractile cycle can proceed. This is the so-called ‘steric blocking model of muscle regulation’ proposed in 1972 based on X-ray diffraction studies of muscle [62,63,64] and later confirmed by electron microscopy [65,66].

The steric blocking model applies to vertebrate skeletal muscles and to many insect muscles. It may also be involved in vertebrate smooth muscles, but, apart from tropomyosin, the actin binding proteins in smooth muscles are different [67]. In other invertebrate muscles there is no troponin regulation, but there is direct regulation through the regulatory light chain of the myosin heads [68]. This light chain in all muscles can be reversibly phosphorylated and this also has a modulatory effect on activation even in vertebrate striated muscles [69,70]. In vertebrate cardiac muscles, C-protein (MyBP-C) can also be phosphorylated and it too can modulate the contractile response [71,72].

**Figure 10:**
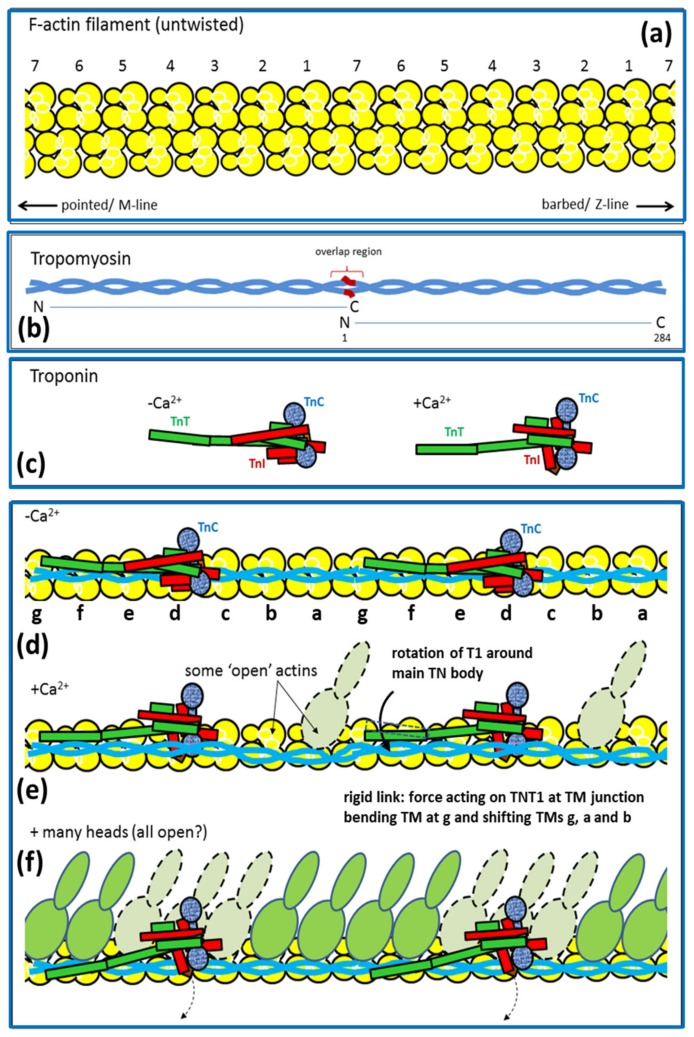
Actin filament structure and regulation: (**a**) An untwisted version of an F-actin filament, (**b**) coiled-coil strands of tropomyosin. (**c**) Troponin structures with and without Ca^2+^ bound. (**d**–**f**) The whole thin filament (untwisted) to illustrate the assembly of the three components in (**a**–**c**) and their changing configurations in different states: (**d**) Off state, no Ca^2+^. (**e**) Closed state, Ca^2+^ bound to troponin alters troponin which shifts tropomyosin strands to where some heads can attach. (**f**) With more attached heads the tropomyosin is pushed over further to the open state and yet more heads can then attach to actin. (**d**–**f**) adapted from Paul et al. [39], with permission.

In Figure 8 the strong AM.ADP.Pi, AM.ADP and AM states are also involved in tropomyosin movement. It is thought that activation is a 3-state process, because attachment of heads in these strong states pushes tropomyosin even further away from its resting position than does Ca^2+^-binding on its own [39,66,73,74], thus making it easier for more heads to attach to actin (see Figure 10). Also there must be a state between the M.ADP.Pi to (A)M.ADP.Pi rapid equilibrium weak-binding state and the strong AM.ADP state, a state which earlier we called the AM.ADP.Pi state, which is the first strongly attached state prior to Pi release. The (A)M.ADP.Pi and AM.ADP.Pi states are known as the pre-powerstroke states. Later we will see that there are even more distinguishable steps in the cycle.

### 2.7. Muscle Mechanics

**Figure 11:**
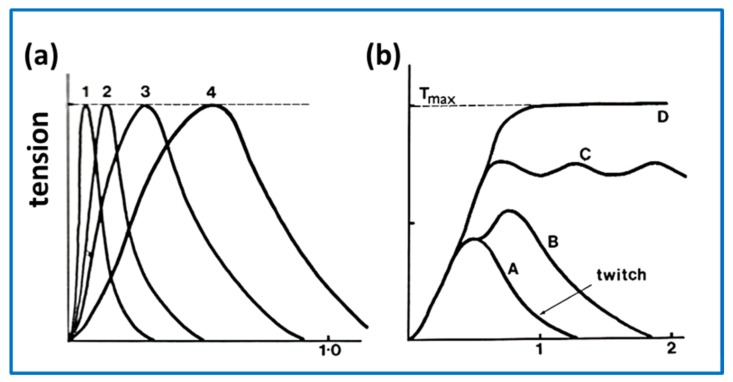
**(a)** Different time scales of the twitch responses in (1) a mammalian gastrocnemius muscle, (2) a mammalian soleus muscle, and (3,4) a frog sartorius muscle at 10 °C (3) and 7 °C (4). (The maximum tensions here have been normalised to the same scale). (**b**) Variation of tension response in a twitch muscle with different stimuli: (A) response to a single stimulus (pulse), (B) response to two closely–spaced stimuli, (C) unfused tetanus produced by repetitive stimulation at moderate frequences, and (D) fused tetanus induced by repetitive stimuli arriving faster than the fusion frequency. T_max_ is the maximum tension, sometimes called P_o_. The horizontal axis is time (seconds). Adapted from [3] after [75].

The amount of tension that a vertebrate skeletal muscle produces depends on the type of muscle and the stimulus (Figure 11). Stimulation of so-called twitch fibres with a short electrical pulse produces a tension twitch in which the tension rises and then decays shortly afterwards, when the stimulus stops. If there are two pulses relatively closely spaced in time so that the second pulse arrives before the first twitch has finished then the tension can build up to higher levels (Figure 11(bB)). If there is a series of closely spaced pulses, then the tension builds up to a steady maximum level known as a tetanus. In ideal conditions, the tetanus is the maximum force that the muscle can produce (T_max_ or P_o_), if it is not subjected to additional stretching. If the length of the muscle is held constant, then it can produce force as above to give an **isometric tetanus**. If the muscle is allowed to shorten under constant load (e.g., with a weight on it) the contraction is said to be **isotonic**. In isotonic contractions, after an initial transient, the shortening velocity is fairly constant with a slope depending on the load. For obvious reasons, muscles shorten relatively fast if the load is high and more slowly for a low load. These are special cases; in reality, the changes in length and the loads that our muscles experience are constantly varying.

**Figure 12:**
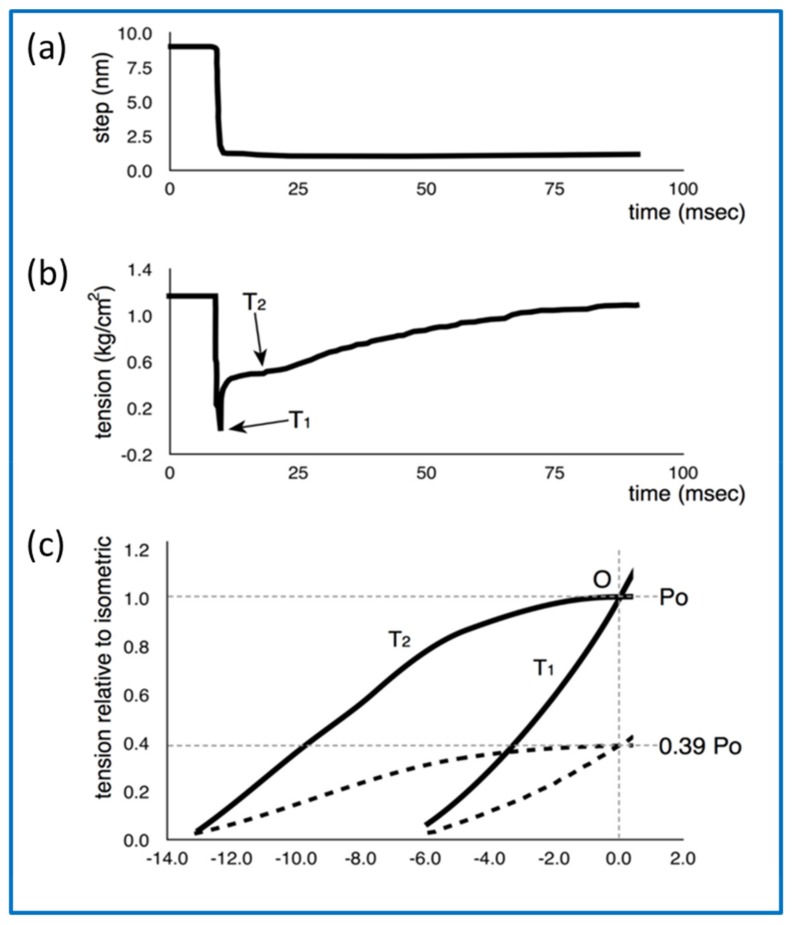
Summary of the results of Huxley and Simmons and their collaborators [76,77,78]. Representation of the experiment showing the tension transient in a fully active intact frog muscle fibre (**b**) after a rapid shortening step (**a**) of about 8 nm. (**b**) shows the point where the T*_1_* and T_2_ tensions were recorded. (**c**) the T*_1_* and T*_2_* plots from experiments as in (**a**,**b**), but for different shortening steps (filament displacement) and shown at two different sarcomere lengths—solid lines full overlap, dashed lines 3.1 µm (0.39% of full overlap). Figure adapted from [79] after [76,77,78].

In 1971, A.F Huxley and R.M. Simmons [76] published a seminal paper in which they reported the responses of single frog muscle fibres to rapid mechanical transients. In particular they stimulated the fibres to produce fused isometric tetani (tension P_o_) and then they rapidly shortened or lengthened the fibre to give sarcomere length changes in the nm range (0 to 100 Å) and complete within about 1 millisecond. Their results, improved by later experiments with faster steps complete in about 0.2 ms [77,78], are summarised in Figure 12 (reproduced from Special Issue Paper [79]). The rapid shortening resulted in a very rapid drop in tension to new tension level T_1_, followed by a slow recovery of tension known as the T_2_ response. After steps of a certain size, the T_1_ tension reduced to zero. This cut-off value on the x axis of Figure 12c occurred after a step size of around 60 Å [76], but in their later work [77,78] they were able to improve the experiment and refine their analysis. Their preferred value for the T_1_ intercept at zero force for frog muscle at around 0 °C came to around 40 Å. In summary, in a fully contracting isometric frog muscle, a shortening step of 40 Å per half sarcomere, complete in about 0.2 ms, would reduce the tension in the system to zero. They also found that if they changed the sarcomere length from full overlap to about 39% of full overlap, the tension dropped to about 39% of its value at full overlap, but the T_1_ intercept on the zero force axis was still around 40 Å (Figure 12c). Huxley and Simmons took this to mean that what they were observing was the behaviour of the myosin cross-bridges attached to actin which were acting as independent force generators, with the number of force-producing heads reducing linearly with a linear decrease in filament overlap (increase in sarcomere length). In other words, both the head stiffness (the T_1_ curve) and the tension recovery (the T_2_ curve) would scale with filament overlap. In 1971 the existence of the lever arm part of the myosin head was not known and it was thought that the whole head might swing on actin or that there might be a shape change of the actin-attached myosin head. With hindsight, under their conditions, we can say that their conclusion would have been that if the end of the lever arm distal to the motor domain of the average attached head was moved by 40 Å, the force in that head would reduce to zero. 

An important aspect of the Huxley and Simmons result was that they thought that the actin and myosin filaments themselves were not changing much in length during the step, so that the only compliant parts of the sarcomere were the actin-attached myosin heads. They estimated that at least 95% of the observed compliance was coming from the heads. 

That this was not the case was demonstrated clearly in 1994 by Huxley H.E and his collaborators [80], and separately by Wakabayashi K. and his collaborators [81]. As detailed in reference [79], there are certain peaks in the low-angle X-ray diffraction patterns from vertebrate striated muscles that are known to come from the actin filaments and others from the myosin filament backbone. 

The positions of these peaks could be measured quite accurately. It was found that the spacings of these peaks increased by a small amount (around 0.2 to 0.3%) on going from a resting muscle to a muscle producing full isometric tension (apart from a 1% or so additional spacing change of the myosin filament due to activation), and then changed again by a small amount if the active muscle was further stretched. This means that the filaments are themselves compliant (like a spring that can be stretched) and therefore that not all of the T_1_ curve seen by Huxley and Simmons and their collaborators [76,77,78] could be coming from the myosin heads attached to actin; some of it was coming from the filaments themselves. It was then estimated that perhaps only one-third of the observed half-sarcomere compliance might be coming from the heads (see [82] for a full review of this). We will return to this later on. 

Figure 12 also shows the slower recovery of tension after the initial shortening step and the location of the measurement where the inflection tension T_2_ is recorded. Huxley and Simmons [76] concluded that the initial part of the recovery process must be from myosin heads already attached to actin being suddenly free to go on to the next attachment configuration in the contractile cycle, thus producing more force. Later in the recovery, attached heads can detach and other heads can attach. It has been known for some time that the ease of attachment of myosin heads to actin depends on the relative positions and orientations of the heads and the actin binding sites. Attachment, which is called stereospecific because the motor domains of the heads have to be in just the right place and orientation in 3D to attach strongly to actin, depends on the point of origin of the heads on the myosin filament and the particular azimuthal position of the actin monomers around the axis of their parent actin filament. This position gradually rotates around the actin filament axis at different axial positions (see Figure 2d and Figure 9). In fact, for a given myosin head, there are patches along a neighbouring actin filament to which it is easier for the head to bind. These have been termed actin target zones or actin target areas [3,15,83,84,85]. It is clear that for some actin azimuths, the ones between the target areas, it is extremely unlikely for a myosin head to bind. In the length step experiment, the relative axial displacement of the myosin and actin filaments produced by the step can move heads that at first could not easily bind to actin into a position further along the actin filament where binding is much easier. These heads can now attach and their force-producing contractile cycle can proceed. As well as reporting on existing attached heads, the later part of the recovery curve will be partly due to these newly attaching heads. Other heads will be pulled to the end of their working stroke, where the probability of detachment is much increased, and the heads will come off actin.

### 2.8. Problems to Be Solved

To summarise so far, many different studies have arrived at good structures for actin filaments, myosin filaments, and the myosin head, and a plausible crossbridge cycle, as in Figure 8, relating the biochemical steps of ATP hydrolysis to the myosin head configurations on and off actin. And we have a good idea, at least at moderate resolution, of how the cross-bridge cycle is regulated by tropomyosin and troponin. But there remain many unanswered questions about how this cross-bridge cycle really works, some of which have been addressed in recent papers or in the present special issue.

Some of these questions are:

(1) Is there direct evidence for the lever arm changing its angle on the actin-attached motor domain when force is actually produced?
(2) Is some force generated simply by the process of head attachment to actin in the initial strong AM.ADP.Pi state before phosphate is released?
(3) Is more force generated during the process of phosphate release?
(4) Is additional force generated during the process of ADP release?
(5) Are the preferred end point lever arm angles different in AM.ADP and AM?
(6) How many of the steps between strong states are regulated by troponin/ tropomyosin?
(7) Are the transition rates between strongly attached states sensitive to the load on the muscle?
(8) Is there direct evidence for the reversal of angular change of the lever arm on the motor domain in the recovery step?
(9) Is the super-relaxed state the only ordered state of myosin heads in relaxed muscle? Or do heads just become disordered on Ca^2+^-activation until they attach to actin to go through the contractile cycle? Or something else?
(10) How much of the compliance of the sarcomere in active muscle is due to the myosin heads and how much to the filament backbones?
(11) What is the maximum extent of lever arm movement produced by the energy released in one ATPase cycle?
(12) How long do myosin heads stay attached to actin in a single cycle in isometric muscle or under different load conditions?
(13) How can the details of the T_2_ recovery response (Figure 11b) be explained?
(14) What are the identifiable changes in the molecular structures of the myosin heads in different muscle states?
(15) Can the elastic properties of the myosin head through the contractile cycle be defined?
(16) In an isometric contraction how many heads are in each state?
(17) In isotonic shortening how do the head populations depend on the load on the muscle?

There are many other questions. We will consider a few of these in the following sections after considering some of the techniques that can be used to study them.

## 3. Methods of Studying the Crossbridge Cycle

In order to address some of the questions listed above there is a plethora of techniques available with enough reliability and in some cases very high spatial or time-resolution. There are also certain muscle types which are favourites for particular types of research. It is not intended to give comprehensive details of these techniques here, but rather to discuss what each technique can do and refer the reader to dedicated texts.

### 3.1. Imaging Methods: Protein Crystallography, Electron Microscopy, Electron Tomography, Single Particle Analysis

**Figure 13:**
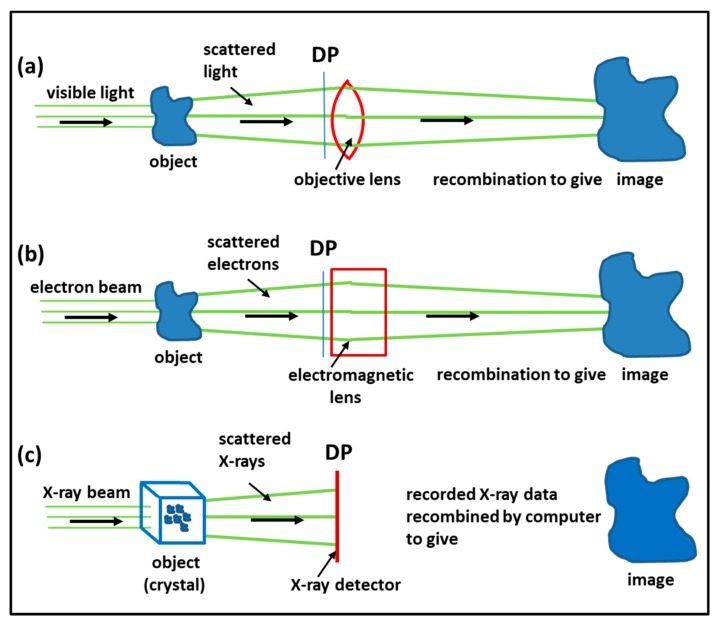
Methods in structural biology: (**a**) Outline diagram showing the steps in an optical microscope. The incoming light interacts with the object and is scattered (diffracted) into spreading beams which enter the objective lens. At the plane marked DP (diffraction plane) the diffraction pattern can be seen. The lens focusses the diffracted beams in such a way that the path length is the same for each of them. These beams recombine to give an enlarged image. Preserving the path length is important since each of the scattered beams of light has a sinusoidally varying amplitude and a relative phase (i.e., are the amplitudes in different scattered beams changing in the same way at the same time—or, if not, what is the lag between them?). If the beams are recombined with the correct amplitude and phase then a faithful image is produced. (**b**) In an electron microscope the same principle applies, but now there is a beam of electrons being scattered and the lenses are electromagnetic. Electrons have wave properties and, once again, they need to be recombined with the correct amplitude and phase to give a good image. (**c**) With X-rays the problem is that X-rays cannot easily be focused; it is difficult to make good X-ray lenses. However, the first part of the procedure can be carried out as with light and electrons. X-ray beams are diffracted and, at the diffraction plane, a detector records the diffraction pattern. The spots on the diffraction pattern have intensities that are the square of the amplitudes of the beams, but, by recording the pattern, the phase information is lost (the phase problem). So, the second half of the process, the recombination of beams to give an image, is done in a computer using various tricks to find the phase information. This is the basis of protein crystallography.

The structural biology techniques have been put together because they all involve some form of imaging of the molecules, molecular assemblies or whole tissues being studied, and many of the fundamental concepts involved in the different methods are related. Some of these concepts are illustrated in Figure 13, where it is seen that light microscopy, electron microscopy and X-ray crystallography all involve recombining beams scattered (diffracted) by the object in such a way that good images are obtained. All of these beams (visible light, X-rays, electrons—and also neutrons—not illustrated) have wave properties defined by an amplitude (how strong is the scattering in that direction) and a phase (are the oscillations in different diffraction peaks changing at the same time or not). The advantage of microscopes is that the effective path length from object to image is the same for all diffraction peaks so they can be recombined, with the relative phases of the peaks intact, to give a faithful image. The problem with protein crystallography is that, since X-ray lenses are difficult to make, the diffraction pattern has to be recorded instead, at position DP in Figure 13, and the amplitude can be determined as the square root of the observed intensities, but the phase information is normally lost. This is the well-known phase problem in protein crystallography. There are different tricks involved in solving the phase problem and, in protein crystallography, the second half of the imaging process is then done in the computer, once the phases have been determined [86,87].

In electron microscopy, the specimens are usually placed on a metal grid, often about 3 mm in diameter and with holes in it like a mesh [3]. Since electrons are easily scattered by air, the whole electron microscope chamber is under high vacuum, so the specimens usually need to be either thin slices of tissue (perhaps less than 100 nm) which is chemically fixed, embedded in resin and sectioned on an ultramicrotome or they can be individual particles or molecules placed on a grid, possibly coated with a thin film, and dried. The study of sections has been highly informative about sarcomere structure, but the resolution is limited to about 50 Å. The three-dimensional distribution of material in cells can be determined either by stacking together information from a ribbon of serial sections, where the axial resolution is limited by the section thickness, or the 3D analysis can be done on one section using the technique of electron tomography. Here a single section is tilted to various angles relative to the electron beam and an image recorded at each tilt angle. A 3D image can then be reconstructed using a kind of back-projection method [88,89,90,91]. In the case of muscle, especially highly ordered muscles like insect flight muscles, the tomographic images show good details of the repeating structures across the sarcomere, and these 3D repeating structures can be ‘cut out’ in the computer and averaged together in a process known as sub-tomogram averaging (Special Issue reference [91]).

For many purposes we need to know molecular structures at the atomic level, which ideally means we need image resolutions of around 1 to 2 Å. The resolution achievable using different kinds of radiation depends fundamentally on the wavelength of the sinusoidal oscillations. In the case of visible light this is around 4000 to 7000 Å and so cell organelles can often be visualised, but not small molecular assemblies. Recently new methods of light microscopy have been developed to extend the resolution limit. This is very informative, but it is still far from what X-rays or electrons can do [92]. X-rays used for protein crystallography or diffraction from whole tissues (see later) often have a wavelength around 1 Å [3], which is why protein crystallography can give really high resolution structural results if the diffracting crystals are well enough ordered. Electrons in electron microscopes have wavelengths that are even shorter, a small fraction of 1Å, so, in principle, electron microscopes ought to provide magnificent resolution. But, until recently, the quality of the lenses in electron microscopes, the efficiency of electron detection, and the deterioration of the specimen by electron beam damage, have limited achievable resolutions to a few Å even for extensive 2D crystals of macromolecules [21,93]. The advantage of using 2D crystals is that the recorded repeating structures in the image can be averaged together; even if each repeat has scattered only a few electrons, as is normally the case with low dose methods, the ‘average’ structure can still be reliable. 

This whole story has changed dramatically in the last few years using new electron microscopes, better electron detectors, cryo-protected specimens and powerful computer algorithms [94]. Now, instead of using 2D crystals to get multiple images of the same object, isolated single protein molecules or molecular assemblies (for example) can be spread onto a grid in a thin layer of water and then the grid can be rapidly frozen to get amorphous ice. The frozen grid can be kept very cold by liquid nitrogen cooling of the speciman stage of the electron microscope, and the specimen viewed in a very high vacuum. The electron image can be recorded, with minimal electron dose on the specimen, using a highly efficient electron detector [95]. The thousands of images of the same molecule or molecular assembly at different orientations around the viewing direction can then be recombined by the relatively new technique of single particle analysis [96] to yield the structure of the specimen with resolutions around 1 to 3 Å [89,94,95,96,97]. It is noteworthy that the 2017 Nobel Prize for Chemistry was awarded to Drs. Jacques Dubochet (Lausanne, Switzerland), Joachim Frank (Columbia University, USA) and Richard Henderson (Cambridge, UK), for “developing cryo-electron microscopy for the high-resolution structure determination of biomolecules in solution”.

In the case of muscle, several of the molecular assemblies of interest are not single molecules, but are extended filaments (e.g., actin and myosin filaments) with repetitive structures along them. In these cases, instead of having many images of isolated molecules or small particles, the individual repeats along the filaments can themselves be cut out in the computer and treated as ‘single particles’. They can then be averaged together in different ways to give good structures for the filaments [98,99]. This is what has been done in references [20,39,51,52,53,54] and is the technique used to generate Figure 4 and Figure 9 of this Review.

### 3.2. Probes: Fluorescence, Fluorescence Resonant Energy Transfer (FRET), Spin Probes 

#### 3.2.1. Fluorescence

**Figure 14:**
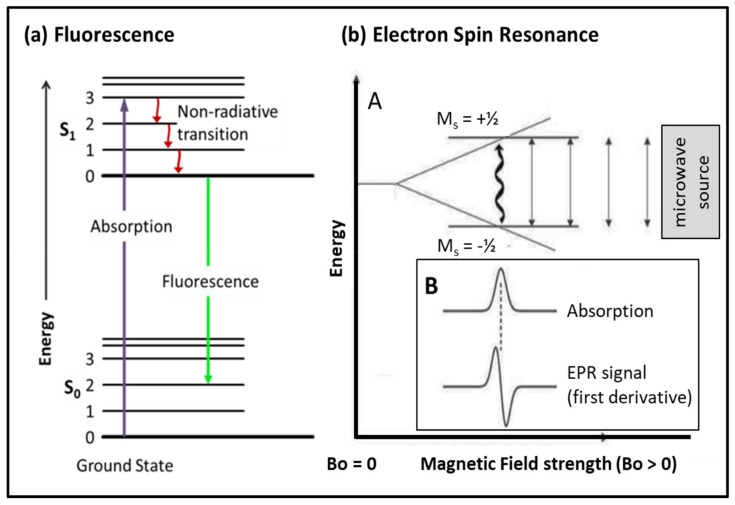
Probes: (**a**) Energy levels in fluorescence probes, (**b**) A: EPR: Spin up and spin down (M_s_ = ± 1/2) energy levels in a magnetic field B_0,_ which become more widely separated the stronger the field_,_ and a microwave source on the right that can flip the spins. Inset (B), the microwave absorption spectrum (top) from the spin flip in A and its first derivative (below). For details see text.

One of the main needs in studying dynamic systems like muscles is to be able to probe specific molecules, for example the myosin head, or myosin binding protein-C (MBP-C), or domains of actin, or troponin and tropomyosin, in such a way as to determine their orientation and/or their mobility under different physiological conditions. Later we will see that some of this can be done by X-ray diffraction, but here we consider the unique contribution that spectroscopy can make. We start with fluorescence. It happens that amino acids like tryptophan are intrinsically fluorescent [100,101,102]. When tryptophan is illuminated by incident radiation of appropriate wavelength (around 3000 Å) electrons in the ground state (level S_0_; Figure 14) are excited to higher energy levels (e.g., upper levels in state S_1_) and then, after some non-radiative jumps between vibration levels within state S_1_, they jump back down to the ground state and re-emit radiation at a lower energy (longer wavelength). If the incident light is polarised, in the case of muscle either parallel (Ppar, intensity Ipar) or perpendicular (Pperp, intensity Iperp) to the fibre axis, then the polarization of the emitted fluorescence is defined as P = (Ipar − Iperp)/(Ipar + Iperp) which is sensitive to the orientation of the fluorophore. It was shown by Aronson and Morales [102] that P is sensitive to the physiological state of the muscle, relaxed, rigor or active, and, although there are tryptophans in other molecules than myosin, Nihei et al. [103] showed that most of the signal was actually coming from the myosin heads.

To be more specific, and to label other things than the myosin head, extrinsic fluorophores can be added to skinned fibres (fibres with their outer membrane (sarcolemma) removed either physically or chemically using detergents) in which some of the known molecules, such as troponin or the myosin light chains or MyBP-C, can be exchanged for equivalent purified proteins carrying extrinsic fluorescent tags, such as IAEDANS [71]. One of the problems of such extrinsic fluorophores is that they can have significant mobility on their parent molecule, even if the molecule itself is fairly static. This mobility can be reduced substantially by the use of bi-functional probes, where the probe is covalently linked to two sites on the parent molecule rather than one (e.g., BR, bifunctional rhodamine [104]).

Fluorescence can also be used to determine distances if there is a donor fluorophore and a receptor within about 100 Å [105,106]. This makes use of what is known as Forster resonance energy transfer (FRET, sometimes called fluorescence resonance energy transfer). This can either use intrinsic fluorophores like tryptophan or added fluorescent labels with mono- or bi-functional binding [105,106]. A possible problem with extrinsic labels is that they may modify the way that a protein normally functions. It is important in the case of muscle to test whether force generation and movement are affected by the labelling.

Exciting developments in spectroscopic methods include luminescence resonance energy transfer (LRET) and total internal reflection fluorescence detection (TIRF [107]). Despite the limited resolution, the latter can ‘detect’ fluorescence from labels on single molecules. Special Issue reference [108] discusses the new technique of ‘time-resolved’ fluorescence resonance energy transfer (TR-FRET) which can directly resolve structural states in the strongly-bound actin-myosin interaction.

#### 3.2.2. Spin Probes

A complementary method to using fluorescence is to use spin probes [109]. The technique, known as electron paramagnetic resonance (EPR) or electron spin resonance (ESR), makes use of the fact that all electrons possess a magnetic moment and will align in a magnetic field. If there is an unpaired electron, this will align either parallel to or antiparallel to the field. The antiparallel configuration (M_s_ = +½) has slightly more energy than the parallel alignment (M_s_ = −½: Figure 14b). A common arrangement is to have the sample, which may be a solution or a skinned or intact fibre, in a strong magnetic field. Electrons are promoted from the lower level (Figure 14b: spin −½) to the upper level (spin +½) by an incident microwave beam of appropriate frequency (energy). With the microwave frequency scanned past the appropriate excitation frequency, the absorbtion spectrum can be detected, recorded (Figure 14b; Panel B) and its first derivative calculated. Alternatively, this can also be done the other way round with the microwave source of fixed frequency and the magnetic field scanned through the optimal field strength.

As with fluorescence, the spin label can be an extrinsic probe such as a nitroxide radical (site directed spin labelling), or it can also be a bifunctional probe to reduce probe mobility, but once again there is a need to check that the probe is not altering the normal function of the host protein. The precise application of EPR depends both on the field strength, the excitation frequency and which signal is recorded. Conventional EPR uses fields of about 3500 Gauss and frequencies in the range 9-10 GHz (X-band). With this set-up, motions with very fast correlation times can be detected (~10^−9^ to 10^−7^ s) using conventional absorbtion. However, muscle proteins have interesting motions in the microsecond to millisecond time window. This window can be studied using saturation transfer EPR (ST-EPR; [109,110,111]). EPR can be used to measure both motions and probe orientations. In addition, measurements of distance can be determined by double electron-electron resonance (DEER) and ab initio high resolution structure determined in muscle fibres by a combination of two-probe BEER with single probe EPR [108,109,110,111]. Bifunctional probes attached to the lever arm of the myosin head can be used to determine the lever arm orientation [111].

### 3.3. Biochemical Kinetics and Caged Compounds

**Figure 15:**
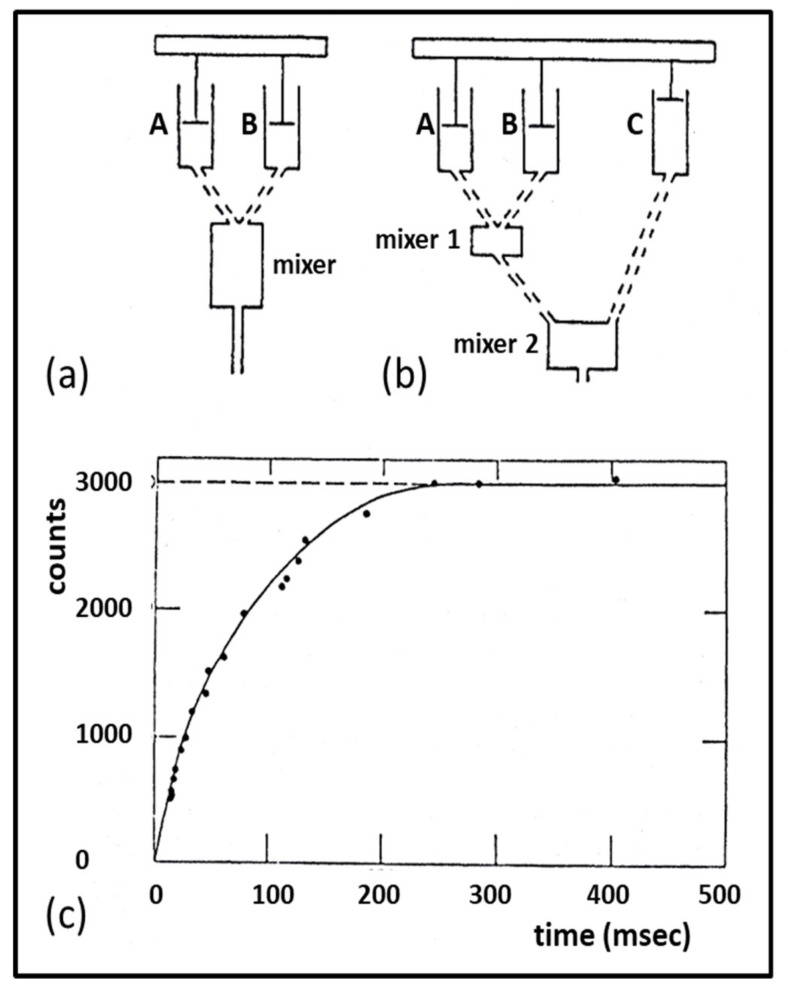
Apparatus used for rapid reaction kinetics: (**a**) stopped flow apparatus, (**b**) quenched flow apparatus. In (**a**) the reactants are held in syringes A and B and are forced into the mixing chamber C within a few msec. The mixing chamber can be monitored optically to follow the progress of the reaction. In (**b**) the reactants A and B, as before, are mixed in a small chamber (mixer 1). The flow is continuous, and the reaction proceeds in the tube between mixer 1 and mixer 2, The reaction is quenched in the mixer 2 chamber by the addition of acid from syringe C. (**c**) Example of a quenched flow result showing phosphate accumulation when rabbit myosin was mixed with Mg-ATP [55]. The initial ATP concentration was 32 M and the magnitude of the Pi burst was 1.2 moles Pi/ mole myosin. (Adapted from [3] after White and Thorson [112]).

**Figure 16:**
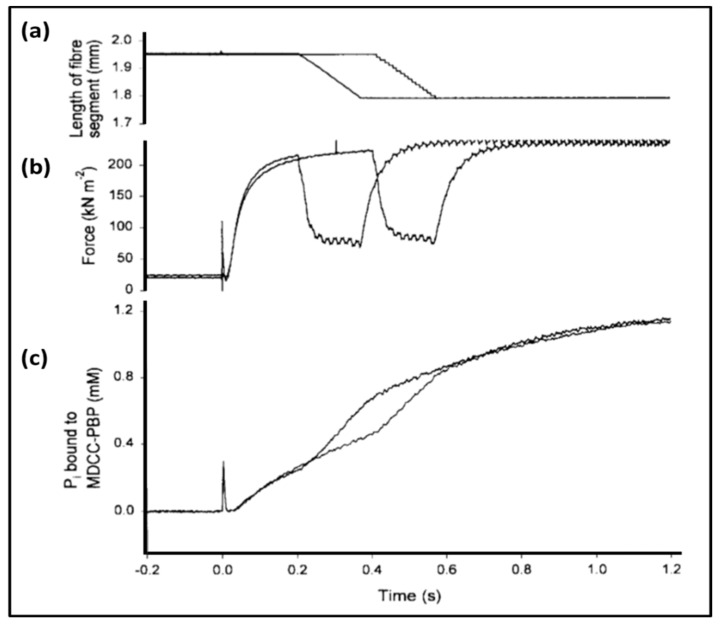
Simultaneous measurement of length (**a**), force (**b**) and phosphate release (**c**) in a single skinned muscle fibre, illustrating the acceleration of Pi release rate during shortening. A permeabilized muscle fibre was mounted between two hooks, one attached to a length-adjusting motor and the other to a force transducer. The Figure shows two consecutive measurements on a single fibre. The fibre was initially at rest length in a rigor solution (no ATP). At zero time a contraction was initiated by the release into a muscle fibre of around 1.5 mM ATP by laser photolysis of caged ATP. (**a**) The length of the fibre as controlled by the motor. At either 0.2 or 0.4 s, the fibre was allowed to shorten by 8% of its length. (**b**) Force measurements: after approximately 0.2 s, the fibre, which initially was prevented from shortening, reached its maximum level of force development. (**c**) The amount of phosphate bound to the Pi-sensor incubated with the muscle fibre. The trace shows clearly that the rate of Pi liberation was increased during shortening steps (adapted from Figure 5 of He et al. [116]).

The biochemical properties of enzymes like myosin, with its substrate of ATP, have been characterised in solution by measuring rate constants, for example between various steps in the acto-myosin ATPase cycle [55,112,113]. All of the transitions in the Lymn-Taylor scheme of Figure 8 are reversible steps, so the relative abundances of the different states around the cross-bridge cycle are determined by forward and backward rate constants. Many of these rates were originally measured in solution using stopped flow or quenched flow methods [55,113] (Figure 15). However, the fact that the acto-myosin II interaction normally occurs in muscle fibres, where the filament geometry will have an effect, means that results from solution may not be directly applicable to what goes on in muscle. For this reason, experiments can be carried out in skinned muscle fibres, for example, using ‘caged’ ATP to initiate contraction. Such ‘caged’ molecules are inactive until unblocked by the application of a light pulse (laser photolysis) to open the ‘gate’ [114]. After that there are additives sensitive to, for example, Pi concentration. Such a Pi sensor, a phosphate binding protein, will bind Pi when it is released from myosin and as a result will fluoresce [115,116]. The recorded fluorescence intensity is a measure of Pi concentration. An experiment of this kind is illustrated in Figure 16, combining mechanical measurements, release of caged ATP and the use of a phosphate binding protein [116].

### 3.4. Time-Resolved X-Ray Diffraction

Apart from the application of X-rays in protein crystallography, so-called low-angle X-ray cameras can be used to record X-ray diffraction patterns directly from whole muscles or isolated muscle fibres, or (at moderate angles) even preparations of isolated actin filaments [3,7,18,19,22,38,57,58,59,62,63,64,80,81,82,117,118,119,120,121,122]. For reviews on low-angle X-ray diffraction theory and results see references [7,119,120]. The basic idea is similar to Figure 13c, except that now the specimen is a whole muscle or a fibre or an oriented gel of actin filaments. Because of the reciprocal nature of diffraction (i.e., the diffraction angle gets smaller the larger is the spacing in the object that is diffracting), since the repeats in actin and myosin filaments are in the tens of nm range (axial repeat 355 Å and 429 Å respectively in vertebrate striated muscles), the angle at which the scattering or diffraction pattern can be seen is very low. Long X-ray cameras (i.e., with long distances between the specimen and detector) are needed to separate the diffraction peaks at the detector. An advantage of low-angle diffraction is that electronic detectors can be used to record the diffraction patterns [123] and these can often be read out within a ms or less, permitting fast time-resolved X-ray diffraction studies of contracting muscles and fibres [57,117,118,119,120,121,122,123,124]. 

The spacings of the observed diffraction peaks can be determined, often with high accuracy, and this is very informative, especially about the size and symmetry of the diffracting objects. The main problem with such diffraction techniques is that, unlike protein crystallography, it is not easy to solve the phase problem. For this reason a common approach is to use what knowledge there is about the proteins involved and then to set up structural models in the computer, using this information, with the molecular organisation in 3D defined by adjustable parameters, for example to define the positions and orientations of particular proteins or protein domains. The parameters are then adjusted in the computer and the predicted diffraction pattern calculated. The goodness of fit between the observed and calculated diffraction patterns can then be assessed, usually using a so-called R-factor see [121], to find which combination of parameters gives the best fit to the observations. This approach can be very powerful [121,122].

Using time-resolved X-ray diffraction with patterns recorded on fast readout detectors [123], the changing diffraction pattern from an active muscle can be recorded on a millisecond or shorter time scale as force is being generated. The steps of the crossbridge cycle can be followed during changes in intensity of various parts of the X-ray pattern and these changes can be modelled to yield time courses of changing populations of cross-bridge states in the contractile cycle. In the right hands, this technique is immensely powerful. But caution is required, since misinterpretation can lead to false conclusions (see discussion in [7,119]). In particular, I believe that those modelling X-ray diffraction data should show clearly that the number of parameters required to fit their model to the data, is significantly smaller than the number of truly independent observations that are available. If this is not done, then the analysis can be assumed to be under-determined and there is no reason why anyone should believe what is being claimed.

The second point (included in the Knupp et al. (2009) analysis [119]) is simply how to assess the information content of a peak like the M3 peak. On its own it simply contains the information that a lump of material of unknown shape is situated at 14.3 nm spacings along the fibre axis. There is no more information than this (other than possibly the extent of the diffracting array). If the peak is sampled by an interference function, then there is additional information about the interference function, but no more information about the shape of the diffracting object. Only by full modelling of all the myosin meridional peaks out to a reasonable resolution (say 2 nm) would one get useful information about the axially-projected shape of the diffracting object. This might then reveal lever arm movements.

### 3.5. In Vitro Methods: Motility Assays, Optical Traps

**Figure 17:**
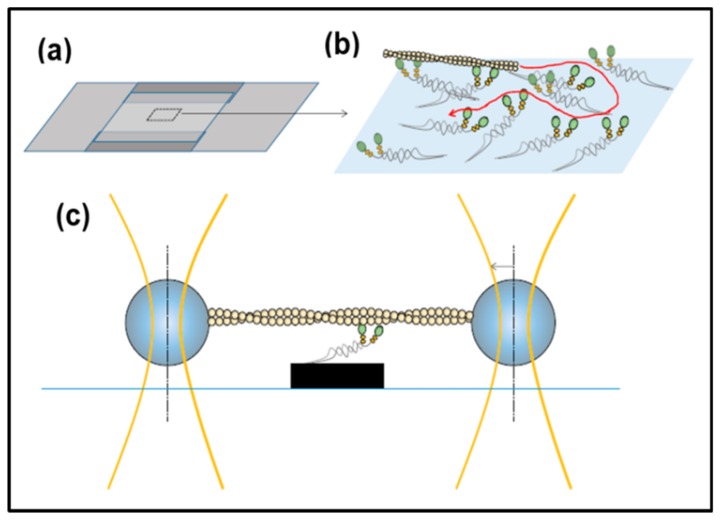
In vitro motility assay and optical tweezers set-up for single-molecule mechanics. (**a**) Flow cell of two cover-slips sandwiched on top of each other via spacers; (**b**) magnified view of rectangular area in (**a**) indicating the principle for the gliding in vitro motility assay with surface-adsorbed myosin heads that propel an actin filament. The curved red arrow indicates a possible filament sliding path; (**c**) schematic diagram of the three-bead optical tweezers assay where an actin filament is suspended between two beads held in optical traps. The filament is then lowered down to allow the actin filament to interact with single myosin motor fragments adsorbed to a third bead or another type of pedestal as indicated here. Reproduced from Special Issue Review [125] with permission.

Apart from studying muscle proteins either biochemically in solution or in the intact fibre, methods have been developed to study the motion or behaviours of isolated filaments or molecules in the light microscope, sometimes with the proteins labelled with fluorescent tags which can be detected, even though not imaged at high resolution. These are called motility assays. For example, isolated myosin heads or heavy meromyosin (two heads and about one third of the myosin rod), can be laid down on a microscope cover slip in an appropriate solution with ATP and calcium and fluorescently labelled actin filaments can be watched as the myosin heads propel the actin filaments across the cover slip (Figure 17a,b). Or use can be made of the photon power in focused laser beams to manipulate small beads in the light microscope; so-called optical trap methods. If these beads are attached to either end of an actin filament (Figure 17c), then this filament can be lowered onto an isolated myosin molecule on a pedestal or on another bead and the force generated by the interaction can be determined by the observed displacement of the actin-attached beads. These are very useful and powerful techniques, which can separate out the effects of different steps between strong states in the cross-bridge cycle, as discussed fully in Special Issue Review [125], where Mansson et al. also compare results from such studies of isolated molecules and filaments with what is found in situ in muscle fibres. The reader is referred to that Review for further discussion of these techniques and appropriate references.

### 3.6. Electron Microscopy with an Environmental Chamber

**Figure 18:**
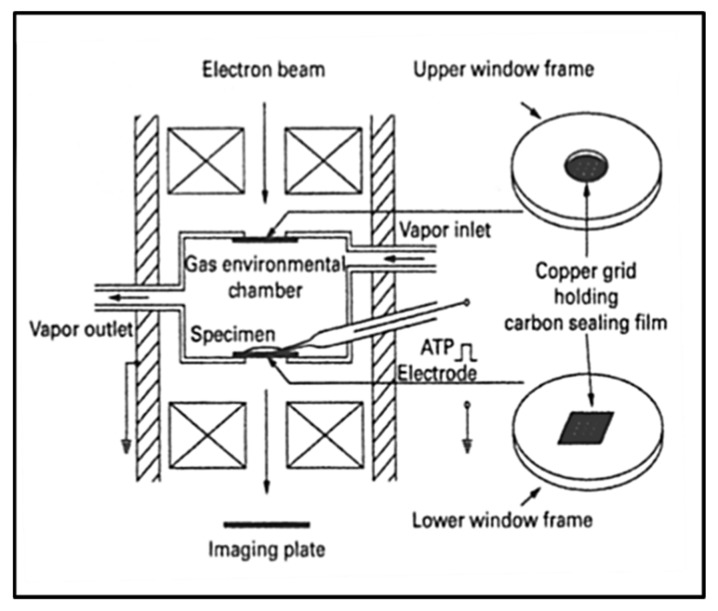
Diagram of the environmental chamber in the electron microscope. The chamber is sealed top and bottom by carbon films through which the electrons can penetrate. ATP is applied to the specimen iontophoretically by passing a current through the ATP electrode, containing 100 mM ATP. Reproduced from Sugi et al. [126] with permission.

An interesting recent development in electron microscopy has been the application of an environmental chamber to visualise domain movements in the actin-myosin system while the proteins are in a hydrated state; the proteins are almost in a physiological environment within the electron microscope (Figure 18). Sugi and his colleagues have been studying the movements of various parts of myosin heads labelled with specific antibodies carrying gold particles, which are visible in the microscope. We will discuss some of their results later, but full details are given in the Special Issue Review [126] by Sugi et al.

## 4. Structural Biology and Mechanics Insights into the Contractile Mechanism

### 4.1. Protein Crystallography and High-Resolution Electron Microscopy

**Figure 19:**
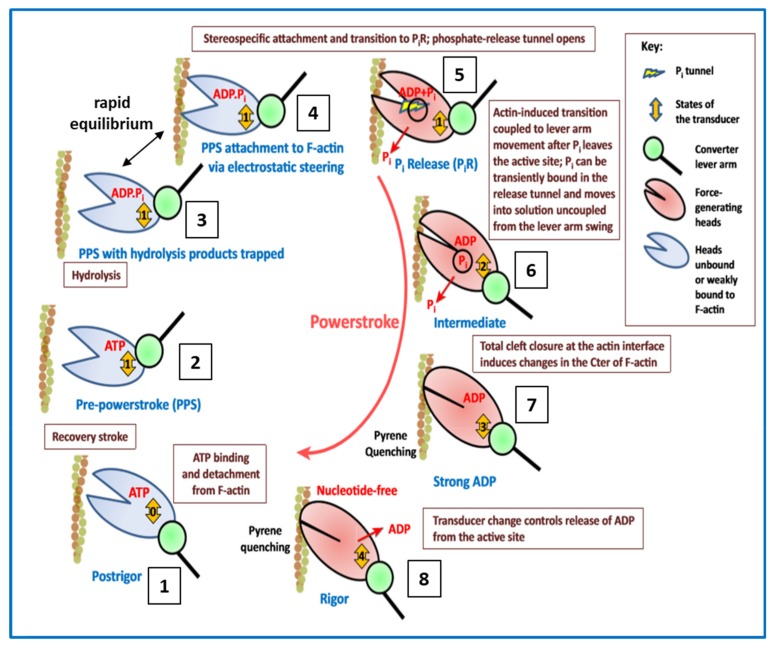
Various stages of the actin-myosin ATPase cycle (cf. Figure 8) as expanded by Houdusse and Sweeney [127]. For details see text. Reproduced from [127] with permission.

Some of the major insights into the contractile mechanism have come from protein crystallographic studies of different myosin heads with various substrates bound. Following the initial ground-breaking study of Rayment and his colleagues [12], the more recent work, reviewed by Houdusse and Sweeney [127], has been particularly notable. In addition, the latest electron microscopy methods have defined high resolution structures for the actin-myosin-MgADP complex at 8Å [128] and the rigor state (no ATP) at 4Å [129]. Figure 19 summarises some of the conclusions of these studies (from [117]). The myosin head motor domain (see Figure 3) has a cleft in it, which, depending on the substrate bound or whether actin is attached, can be closed or open, or partially open. This is called the actin-binding cleft. Also at the heart of the motor domain is a so-called transducer (mainly a 7-stranded β-sheet) which changes conformation to accommodate MgATP, when the sheet is flat and strained. If no nucleotide is bound, the sheet is curved and relaxed. There is an intermediate state of the transducer when the motor domain binds actin and MgADP with high affinity, as in the major force-generating part of the cross-bridge cycle.

When ATP binds to the myosin head, the products ADP and Pi are rapidly produced, but these products are not readily released until the head binds to actin. In Figure 19, the left side shows head states off actin or weak-binding to actin, whereas the right hand figures show strong head states on actin. In each head image the actin-binding cleft is on the left and the converter and lever arm are on the right. The numbered double arrow shows the state of the transducer. The progress of Pi release is not straightforward in that its exit via the ATP binding pocket is blocked by the MgADP that is still bound, so there must be another exit (a back door) in the motor domain for the Pi to be released from. Such a back door has been seen in crystals of myosin VI [130], but not in heads bound to actin. In the non-attached states this Pi exit is blocked.

A recent disagreement has been about whether force-production occurs before or after Pi release [127,130,131]. To quote Houdusse and Sweeney [127] ‘The debate centres on whether the powerstroke gates phosphate release, or whether phosphate release gates the power stroke.’ Some recent studies have suggested that force-generation occurs first. However, protein crystallography studies suggest that the Pi released from the ATP-binding pocket and exiting through the back door may get trapped there and may only be fully released and detectable at a later time. In this case, Pi release from the ATP binding pocket might be an early event that triggers the power stroke, but Pi release into the sarcoplasm would only be detectable after a delay. 

The cycle in Figure 19 shows heads either on or off actin, with the actin-binding cleft either open or closed, with the lever arm/converter domain either up or down and with the transducer in several different states. For example, the binding of ATP to the rigor head (state 8 in the Figure) gives the AM.ATP state which rapidly detaches from actin to give the M.ATP post-rigor state (1) in which the lever arm is still down. In this scheme, resetting of the lever arm occurs before ATP hydrolysis when there is a change of state of the transducer to give conformation (2). Hydrolysis then occurs (2 to 3), and the resulting M.ADP Pi state can then interact with actin in a rapid equilibrium, weak-binding, state during which the attached head may explore the actin surface to find a suitable, strong binding site. When it finds this, there is stereospecific labelling of actin by the head, the actin-binding cleft partially closes, and Pi release is triggered to give the AM.ADP state (6) with the actin-binding cleft almost closed and the lever arm tending to swing down. Further closure of this cleft may be associated with a change of state of the transducer (from 6 to 7). ADP release then fully closes the cleft and further lever arm rotation (at least in some myosins) can occur (from 7 to 8), after which the cycle can start again.

### 4.2. Low-Angle X-ray Diffraction: Static Muscle States and Time-Resolved Studies

One of the main driving forces in muscle studies, for obvious medical reasons, is to understand in detail how heart muscle works. One of the main aspects of heart muscle that enables it to function as it does is that it pulls harder when it is stretched. This is the well-known Frank-Starling law [132]. So, what might change as the sarcomere length increases? An obvious possibility is that perhaps the actin filament becomes more switched on with stretch because the tropomyosin shift is enhanced [132]. But an unexpected finding in the Special Issue paper by Eakins et al. [133], not on cardiac muscle, but on bony fish muscle, was that the same muscle put into rigor at two slightly different sarcomere lengths (2.2 and 2.5 µm) showed myosin head attachments to actin which were quite different. One was not just a scaled down version of the other, as might be expected because of the reduced filament overlap at 2.5 µm. Could this kind of effect, if it occurs in heart muscle, be associated with the Frank-Starling Law? (See also [134])

Recent time-resolved X-ray diffraction studies of contracting muscle [124] have attempted to model the varying time-courses of several X-ray reflections on the equator of the diffraction pattern in terms of the occupancy of various states and their likely diffraction contributions. The cross-bridge cycle used for the modelling was less complicated than that in Figure 19, but it included groups of states with contributions to the equator which were likely to be similar. So there was Group (a), heads off actin, Group (b), heads in the weak-binding and transiently-attached pre-powerstroke states, Group (c), strong and rigor-like heads (i.e., states on the right in Figure 19), and Group (d), heads off actin before hydrolysis and the transition back to Group (a). It was found that quite a good fit to the data was with occupancies of (a) and (d) together 48%, (b) 20% and (c) 32%. Most notable here is that of state (b) which has a significant number of heads in the weak-binding states.

Some of the advantages of using insect flight muscle as a specimen for both static and time-resolved X-ray diffraction studies are described in the Special Issue Review by Iwamoto [135]. Even diffraction from single sarcomeres and from muscle cross-sections can now be achieved. Some remarkable time-resolved experiments include those from a whole Drosophila (fruit fly) actually in the synchrotron X-ray beam and using its flight muscles to do oscillatory work [136].

### 4.3. Further Aspects of Muscle Mechanics

Above we discussed the Huxley-Simmons (1971) [76] experiments on the tension transients resulting from rapid step changes of length of frog muscle fibres, and their improved results with better experimental set-ups [77,78]. These experiments have formed the backbone of the thinking of researchers on how muscle works ever since. However, in a recent paper in this Special Issue, the interpretation of the T_1_ curve has been questioned [79]. What was done was to model in the computer all the known compliances in the sarcomere as Hookean springs with different stiffnesses depending on whether it was the myosin filament backbone, the actin filament, the cross-bridges and even titin. What was found was that, instead of the myosin cross-bridges accounting for about one-third of the half-sarcomere compliance, the observed X-ray spacing changes seen by Huxley et al. [80] and Wakabayashi et al. [81] could be explained almost entirely by the compliances of the myosin and actin filaments alone—the exact value of the cross-bridge stiffness made very little difference, as long as it was more than around 0.4 pN/Å. In this case the apparent cross-bridge stiffness was much higher than had been thought previously. Instead of being around 0.15 to 0.2 pN/Å [82], it appeared to be significantly greater than 0.4 pN/Å and possibly much higher. But it was also realised that, as well as the strong-binding heads, the weak-binding bridges must also be contributing to the instantaneous stiffness seen in the T_1_ curve. In fact, it is under the very fast shortening conditions of the T_1_ curve measurements that the weak-binding heads would show stiffnesses as great as or even more than the stiffnesses of rigor bridges [56].

The problem with such a high cross-bridge stiffness, if it applies to the strongly attached, force-producing states, is that the known amount of energy available from ATP hydrolysis, assuming about 50% efficiency, would only generate a very small swing of the lever arm (about 30 to 35 Å) before the lever arm tension had dropped to zero. But the best estimates of the throw of the lever arm is about 100 Å, which would require a cross-bridge stiffness of only around 0.1 pN/Å. What can be going on?

Happily, there is a way out of this dilemma. The suggestion in [79] is that by far the biggest cross-bridge effect on the T_1_ curve is from the weak binding bridges. So the high stiffness based on the T_1_ curve would in this case not be primarily reporting the behaviour of the strong bridges. It was suggested that the effect of the first strong-binding state associated with phosphate release could be to release the lever arm so that it can now act as a softer spring with a stiffness of only 0.1 pN/Å, a spring that can give each head a throw of 100 Å before its tension drops to nothing. With this scheme, the Huxley-Simmons observations would be entirely consistent with conclusions from other experimental approaches about the lever arm movement that can result from a single ATP turnover. Note that, with this scenario, during the T_1_ step, both the weak-binding and strong-binding heads would be contributing to what is seen and the tension change will still be reporting what the strong heads, the weak heads and the myosin and actin filaments do. But the stiffness (slope) of the T_1_ curve will be dominated by the compliance of the filament backbones and the stiffness of the weak-binding heads and their effect on any changes in muscle length, because the weak-binding heads (in this model) are much stiffer than the strong-binding heads. Note that, as yet, the idea that the strong binding states might be less stiff than the weak binding states is only a conjecture; it is yet to be tested properly.

The T_2_ curve of Huxley and Simmons [76] was originally thought to be reporting the behaviour of the strong bridges as they rebuilt the tension in the filaments and redistributed themselves amongst the available strong states. There is no reason, as yet, to doubt that this is still the case, but, once again, interpretation of various results on the T_2_ tension recovery processes also needs to be clarified.

The review by Ranatunga [137] discusses how the mechanics of the crossbridge cycle are affected by changing the temperature, sometimes using rapid T-jumps. Temperature has a marked effect on the level of tension produced by most muscle types. To quote Ranatunga: ‘Analysis showed that a T-jump enhances an early, pre-phosphate release step in the acto-myosin (crossbridge) ATPase cycle, thus inducing a force-rise. The sigmoidal dependence of steady force on temperature is due to this endothermic nature of crossbridge force generation. During shortening, the force-generating step and the ATPase cycle are accelerated, whereas during lengthening, they are inhibited. The endothermic force generation is seen in different muscle types (fast, slow, and cardiac).’

### 4.4. The Electron Microscopy of Myosin Heads

Two reviews in the Special Issue discuss the application of quick-freeze electron microscopy to capture transient sarcomere structures and visualise them in the electron microscope. In one [91], by Taylor and his colleagues, the quick freezing of intact fibres (in this case insect flight muscle) was followed by low-temperature embedding (freeze-substitution), sectioning and electron tomography to reveal the 3D distribution of density within the sections. Because of the beautiful 3D regularity of insect flight muscles [83], this technique is particularly effective. Once a tomogram had been obtained, structures which repeat across the muscle section, because of the unique order in this muscle, could be ‘cut out’ in the computer and averaged together using sub-tomogram averaging [90,91]. In this way, a variety of images of myosin heads attached to actin have been visualized and attempts made to fit the known crystal structure of the myosin head into the density.

In another approach [138], Katayama and his colleagues have combined quick freezing with motility assays on a mica surface and then produced deep-etch, shadowed, replicas using something like the Heuser technique [139]. They have then isolated individual electron microscope images of myosin heads on actin filaments and have tried to correlate these with the known myosin head structures in different states as they would appear after heavy metal shadowing. They claim to recognize different known head shapes and even some previously unknown ones.

**Figure 20:**
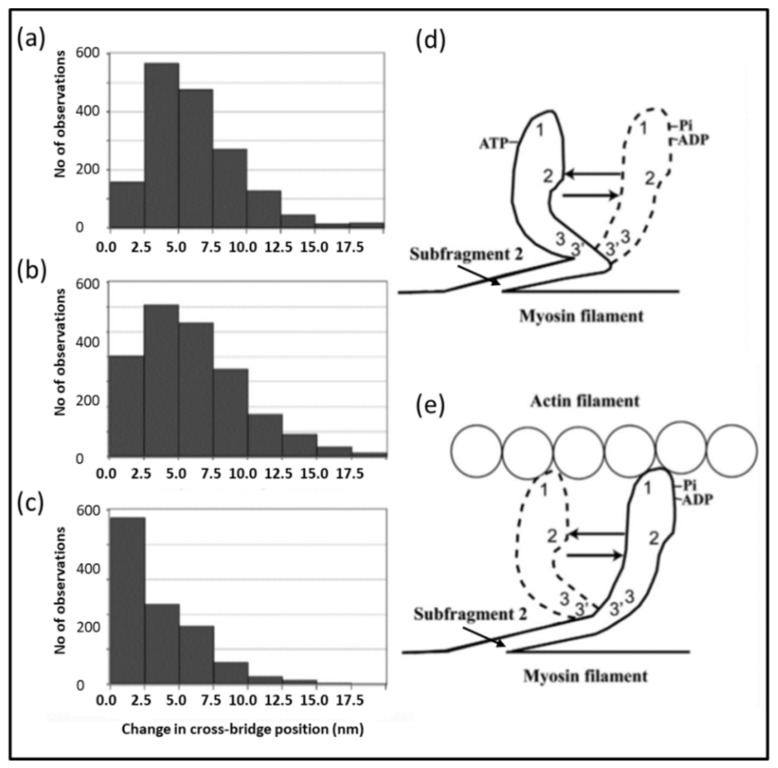
Results from use of the electron microscope environmental chamber to study myosin head conformations under different conditions. (**a**–**c**) Histograms showing the amplitude distribution of ATP-induced myosin head movement in the recovery stroke in the absence of actin at the distal (**a**), and the proximal (**b**) regions of the myosin head motor domain, and at the two regulatory light chains located at the proximal lever arm region of the myosin head (**c**). (**d**,**e**) Diagrams illustrating the ATP-induced changes in the myosin head configuration in the absence (**d**) and in the presence (**e**) of actin filaments. These are based on histograms such as (**a**–**c**) and similar histograms for the movement in the presence of actin. In (**e**) the heads start in rigor and then ATP is added. The heads are thought to come off actin, hydrolyse ATP, rebind to actin and then go through a power stroke. Sites 1 and 2 in (**d**,**e**) are the distal and proximal labelling positions discussed in (**a**,**b**), and sites 3 and 3′ are the light chain labelling positions discussed in (**c**). Adapted from ref [126] with permission.

In the totally different and heroic approach by Sugi and his colleagues [128,140], discussed earlier (Figure 18), some results from their studies of hydrated actin-myosin samples in the environmental chamber of an electron microscope are at the limit of what can be achieved and are tantalizingly provocative (Figure 20). They used gold particle-labelling to identify myosin head positions and structures through labels attached via antibodies to different parts of the myosin head. In this way, they believe they have seen evidence of reversible myosin head movements in the presence of ATP, but in the absence of actin, away from the myosin filament bare zone and in opposite directions on opposite sides of the bare zone. They take this to be the resetting stage or recovery stroke (1) to (2) in Figure 19.

In the presence of actin and with three different antibodies labelling the heads on either the motor domain or the light chains they believe they can actually see a change in myosin head shape consistent with the kind of lever arm movements that are illustrated in the conventional cross-bridge cycle (Figure 20).

### 4.5. Conclusions about the Crossbridge Cycle

**Figure 21:**
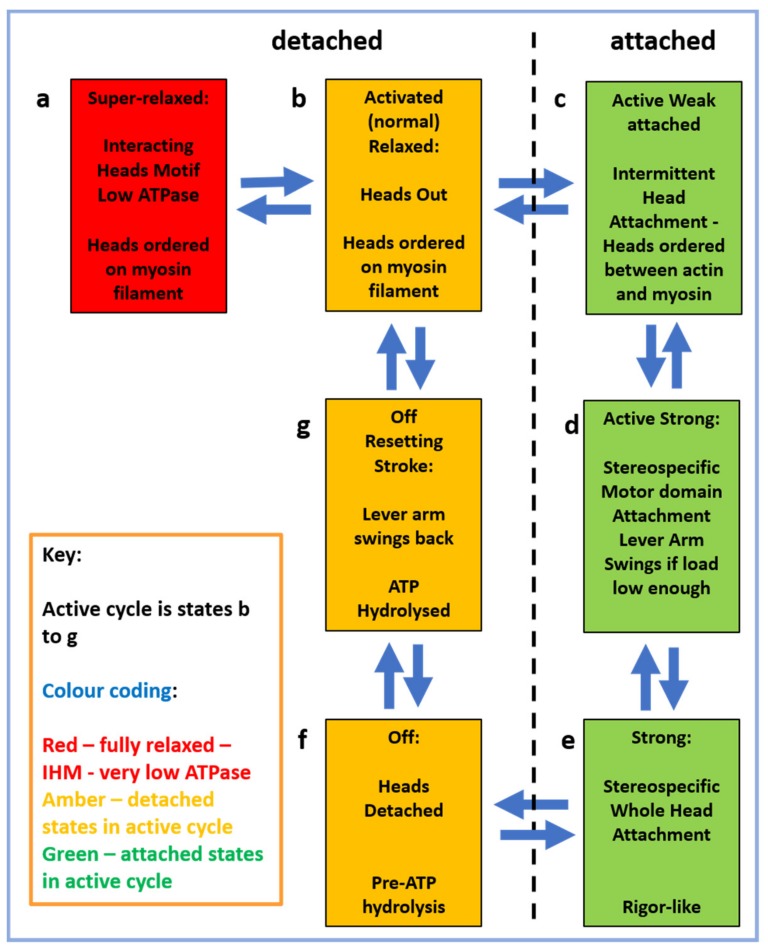
Schematic diagram showing the different states that have been identified from electron microscopy and low-angle X-ray diffraction studies: (**a**) the fully relaxed (super-relaxed) state; (**b**) the normal (activated) relaxed state in intact muscle; (**c**) the attached part of the weak-binding state where heads are in a rapid detached-attached equilibrium; (**d**) the strong states during which the lever arm is supposed to swing axially towards the rigor state (**e**); (**f**) the rapid detachment caused by binding of ATP to the rigor-like heads in (**e**); (**g**) the resetting state where ATP is hydrolysed and the lever arm returns towards its configuration in resting muscle. All transitions are reversible, but some are more likely in the forward direction around the cycle. For discussion see text.

With results from a variety of techniques available it is possible to put together features in the crossbridge cycle other than those depicted in Figure 19 which was purely based on results from protein crystallography. These results are illustrated in Figure 21.

Firstly, we can think about resting muscle. We have seen that many electron microscopy studies of isolated myosin filaments using single particle analysis [50] have come up with structures such as that in Figure 4 which show heads in the interacting head motif conformation (Figure 6). However, X-ray diffraction patterns from normal resting muscles show that the heads are in a different conformation on myosin filaments in the intact A-band filament lattice of bony fish muscle [22], insect flight muscle [22] and mouse Soleus and EDL muscles [141]. To quote from the Special Issue paper by Ma et al. [141]: ‘When the myosin inhibitor blebbistatin is used to inhibit force production, there is a shift towards a highly quasi-helically ordered configuration that is distinct from the normal resting state, indicating there is more than one helically ordered configuration for resting cross-bridges’. The quasi-helically ordered configuration is taken to be the super-relaxed state and the normal (activated) relaxed state for bony fish muscle is modelled to be as in Hudson et al. [121] and that for insect flight muscle is modelled as in AL-Khayat et al. [122]. It remains to be seen what controls the transitions between the super-relaxed and normal (activated) relaxed states.

The first attached state of the myosin heads is thought to be the weak-binding state where the heads are in a very rapid equilibrium between attachment and detachment and the head stiffness in muscle mechanics experiments then depends on how fast the muscle is pulled. During normal contractions the weak heads will be on and off so fast that they contribute little to the muscle stiffness [79]. What do we know about the structure of this state? The study by Eakins et al. [142] found that in X-ray diffraction patterns from active bony fish muscle there is a remnant of the myosin layer line pattern that is not just a reduced version of the resting layer line pattern, as would be expected for example if some fibres in the muscle were not active. What is seen is a different distribution of intensity along the layer lines, consistent with heads still being myosin-centred, but perhaps with variable azimuthal shifts to make the heads point towards actin where they can take part in the weak-binding attachment to actin. Ma et al. [14] also saw residual myosin layer lines in their patterns from active mouse muscle.

Turning now to the attached states of the heads on actin (the right column in Figure 21), there is good evidence for actin attachment both from the actin layer-lines which are enhanced due to the extra mass labelling actin and at the same time the equatorial intensities are such that the 10 peak decreases and the 11 peak increases in intensity in patterns from active muscle, consistent with myosin head mass moving from the myosin filaments towards the actin filaments. As mentioned above, the detailed analysis by Eakins et al. [124] using the first few peaks on the equator from bony fish muscle showed that the time-courses of the intensity changes in patterns from actively contracting bony fish muscle were consistent with something like 20% of the heads being in the weak-binding and pre-powerstroke states, 32% of the heads being in strong states and the rest of the heads being off actin.

In addition, the meridional M3 peak at 14.3 nm, that in patterns from resting muscle comes from the head configuration on the myosin filaments (state (b) in Figure 21), has been shown by Eakins et al. [142] to have two components in patterns from active bony fish muscle, one which is narrow across the meridian being attributable to heads in the weak-binding state and a peak that is much broader across the meridian which is consistent with coming from strongly attached heads on actin, heads that are stereospecifically-attached to actin. More analysis is needed to get details of these strongly-attached head states. What is particularly needed is structural evidence that the lever arm really does swing during the crossbridge cycle in stage (d) of Figure 21. It was claimed in papers such as [143] that changes in the M3 meridional peak intensity show how the lever arm swings on actin, but it was shown unambiguously by Knupp et al. [119] that this conclusion is unreliable—the same data can be explained in other ways. Most people believe that lever arm swinging occurs, but we still need definitive evidence on how much lever arm swinging occurs at various stages of the cross-bridge cycle.

## 5. Mutations in the Actin-Myosin Contractile Apparatus: Muscle Diseases

**Figure 22:**
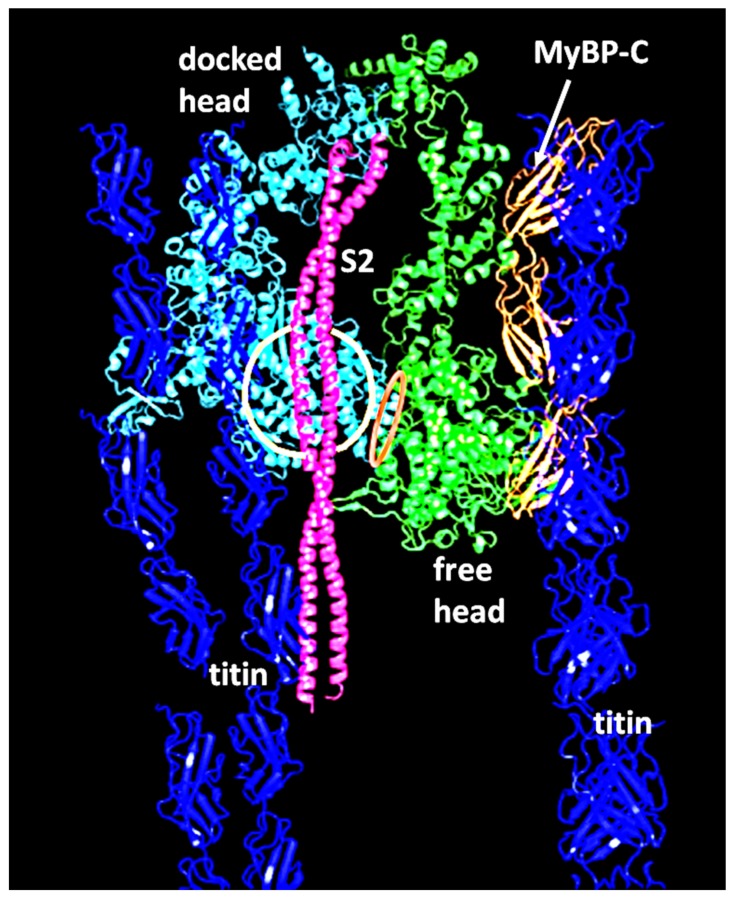
Part of the IHM structure in Figure 4 [21] as detailed by Marston [144]. Strands of titin are shown in dark blue, three domains of MyBP-C in orange, the myosin subfragment-2 (S2) in pink, and the myosin heads in pale blue (docked head) and green (free head). The myosin mesa on the docked head is circled in orange and the free head converter domain interacting with the docked head is indicated by a red ellipse. Adapted from Figure 2c of Marston [144] using structural data from [20].

Three of the Special Issue articles address the ever-progressing understanding of how mutations in some of the contractile proteins are associated with specific diseases [144,145,146]. There are several myopathies in skeletal muscle, such as nemaline myopathy, cap myopathy (Cap), congenital fibre-type disproportion (CFTD) and distal arthrogryposis, that are all associated with mutations in the contractile proteins and are all being actively studied. But by far the biggest concentration of effort is into the cardiomyopathies, the heart diseases, which are a major cause of death. These include hypertrophic cardiomyopathy (HCM), dilated cardiomyopathy (DCM), atrial fibrillation, atrial-septal defect and left ventricular non-compaction. DCM, for example, to quote Marston [144], ‘accounts for 30–40% of heart failure and is the second commonest cause (of death) after coronary artery disease’. The review by Marston discusses the relationship between the phenotype of different diseases and their genotype. And this is not straightforward. The observed mutations in these diseases are largely in myosin heavy chain genes, myosin light chain genes, actin genes, tropomyosin genes and MyBP-C genes. But, for example, to quote Marston [144] again, ‘over 1500 mutations in nine genes have been linked to the single phenotype of hypertrophic cardiomyopathy’. Of particular note is that there is part of the myosin head motor domain, the so-called myosin mesa, that is involved in the interacting heads motif (IHM) structure (Figure 6) and is a hot spot for mutations. Mesa literally means table (Spanish & Portuguese) but has come to mean ‘flat-topped’, often referring to mountains. On the myosin head it refers to the flat face of the docked head that interacts with myosin S2 in the IHM structure, as illustrated in Figure 22 from Marston [144].

To quote Marston [144] again ‘The IHM probably involves additional proteins apart from myosin; MyBP-C is one of the most likely participants. Cardiac MyBP-C is an extended protein consisting of ten domains and it has been established that the N-terminal C0-C2 (domains) lie over the myosin mesa (Figure 22) and also contact the proximal S2 and MLC2 (regulatory light chain).’ He continues: ‘Furthermore this is a regulated structure with IHM stability being modified by MLC2 and MyBP-C phosphorylation’ (see references in [144]). Later he says ‘A final piece of evidence to support the IHM hypothesis is the development of an anti-HCM drug MYK-461 (now known as Mavacampten), found by high throughput screening. It has been shown to be effective both in vitro and in vivo in reversing most of the symptoms of HCM [147,148,149]. A recent study has shown that the mode of action of Mavacampten is to stabilize the IHM structure and enhance the super-relaxed state of myosin [149].’

**Figure 23:**
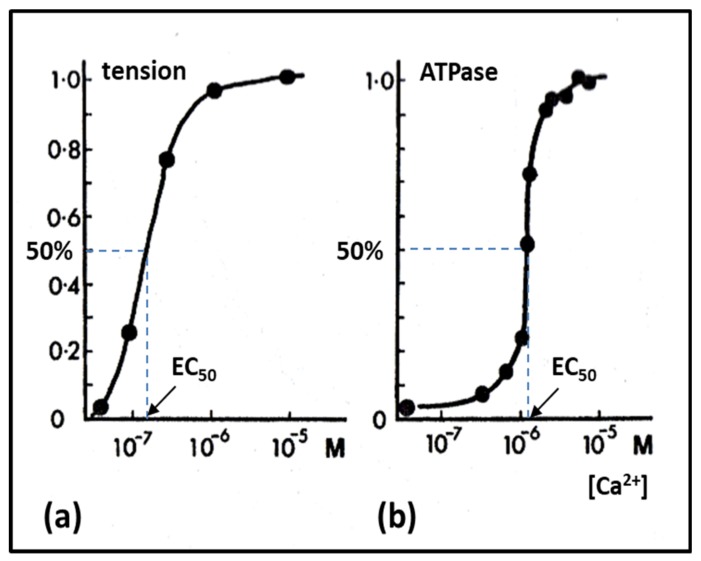
Examples of measurements of (**a**) tension and (**b**) ATPase as a function of the concentration of calcium [Ca^2+^]. Both follow a sigmoid curve centred between 10^−7^ and 10^−6^ M Ca^2+^. Also shown is the EC_50_ value where the parameter in question (e.g., tension or ATPase or other) has reached 50% of its peak value. Shifts of the sigmoid curve to the left or right indicate increased or decreased sensitivity respectively. (Adapted from Squire [3] after [150,151]).

The Special Issue review by Vikhorev [145] discusses how different cardiomyopathies affect the actual functioning of heart muscle. Some key parameters here are the maximum force that the muscle can develop (they call this F_max_), the calcium sensitivity (EC_50;_ see Figure 23), and the rate of relaxation (k_LIN_). Interestingly, the F_max_ for HCM muscles is much reduced, whereas for DCM samples it is normal. The EC_50_ values for both DCM and HCM are reduced to lower Ca^2+^ concentrations (increased sensitivity). This EC_50_ change may be due, in some cases, to the measured reduced phosphorylation of TnI and MyBP-C in hearts with cardiomyopathy. Treatment of these samples with protein kinase A (PKA) to increase phosphorylation of both TnI and MyMP-C largely restored the calcium sensitivity to control values.

One of the important features of cardiac muscle is that it pulls harder when it is stretched (the Frank-Starling Law [134]). This length-dependent activation was found to be reduced in both HCM and DCM muscles, whereas F_max_ was lower for HCM, but normal with DCM. Phosphorylation with PKA restored normal function in the case of DCM, but not for HCM samples. As a summary, to quote Vikhorev et al. [146]: ‘Although an increase in Ca^2+^-sensitivity is the main feature of all cardiomyopathies and heart failure, dephosphorylation of TnI was the major factor responsible for this increase. Therefore it is essential here to differentiate the direct effect of mutations in proteins on myofibril contractility from other secondary effects.

Turning now to the paper by Borovikov et al. [146], they studied muscle dysfunction associated with point mutations in a particular tropomyosin (Tpm3.12). Multiple isoforms of the Tm family are generated by alternative splicing of three tropomyosin genes, and their expression is highly regulated. Extensive spatial and temporal sorting of Tm isoforms into different cellular compartments has been shown to occur in several cell types. Borovikov et al. [146] studied various mutations associated with specific skeletal muscle abnormalities (listed earlier); E173A, R90P, E150A and A155T. They found that E173A, R90P and E150A all produced an unusually large displacement of tropomyosin towards the inner domains of the actin filament, and an unusually high proportion of strongly attached bridges at both low and high Ca^2+^ levels (compare Figure 10). This is a characteristic of CFTD (congenital fibre type disproportion). On the other hand, the A155T mutation caused a decrease in the number of strong heads at high Ca^2+^ levels, typical of the mutations causing Cap (cap myopathy). They conclude: ‘Consequently, in order to choose targets when developing a strategy for treatment of various congenital diseases, it is necessary to have information about the impact of the disease-associated mutation on the behaviour of the regulatory and contractile proteins.’

All three of these studies [144,145,146] show how rapidly the understanding of different myopathies is progressing. And this is all because of the availability of atomic resolution structures for the main contractile proteins (e.g., myosin, actin, tropomyosin and MyBP-C), evidence on the interacting head motif [21], and evidence from biopsies of patients with known myopathies about specific mutations in some of these proteins. And all of this arose from original fundamental research into the steric blocking mechanism of regulation [62,63,64,65], the structures of actin filaments [38,39,128,129] and of human cardiac myosin filaments [20], and mechanical and biochemical studies of muscle fibres [3,55,56,73,74,75,76,77,78,79]. When in 1986 I wrote the monograph entitled “Muscle: Design, Diversity and Disease” [152], the last part of the book was about muscle diseases. At that time it was possible to describe some of the ultrastructural features of muscles from patients with different diseases, but there was little prospect at that time of being able to reassure these patients about possible treatments, mainly because of our lack of knowledge of the fundamental causes of the diseases. Today that situation has radically altered. Although the major muscle problems, Duchenne and Beckers Muscular Dystrophies, are associated with non-contractile proteins (in this case dystrophin and utrophin [153,154,155]), and gene and other therapies are being developed for those diseases, defects in many other important diseases, including the cardiomyopathies, are related to the proteins in the contractile apparatus, and here also possible treatments, such as that involving Mavacampten or others using gene therapies, are on the horizon [156]. It is a wonderful thing that fundamental muscle studies, often carried out originally simply to understand how muscle works, are now, as many of us had hoped, leading to real medical benefits.

## 6. Conclusions

Research into how muscle works has progressed in leaps and bounds since around the 1950s, but there is a great deal that we still do not know. The papers and reviews in this Special Issue have provided insights into some of the questions listed earlier, but these questions are not fully answered, the list is by no means complete, and there is still much more to do. Since muscle and other actin-myosin based motile systems are so fundamental to life, it is imperative that we have a better understanding of how we move. And, since many of the advances in our understanding of heart disease and skeletal myopathies have been based on basic research into muscle structure and function, it is imperative that funding for basic muscle research continues to be forthcoming. Many lives depend on it.
